# Bayesian modeling with locally adaptive prior parameters in small animal imaging

**DOI:** 10.3389/fnume.2025.1508816

**Published:** 2025-03-04

**Authors:** Muyang Zhang, Robert G. Aykroyd, Charalampos Tsoumpas

**Affiliations:** ^1^ Department of Statistics, School of Mathematics, University of Leeds, Leeds, United Kingdom; ^2^ Department of Nuclear Medicine and Molecular Imaging, University Medical Center Groningen, University of Groningen, Groningen, Netherlands

**Keywords:** Bayesian modeling, inhomogeneous parameter, image processing, Markov random field, Markov chain Monte Carlo

## Abstract

Medical images are hampered by noise and relatively low resolution, which create a bottleneck in obtaining accurate and precise measurements of living organisms. Noise suppression and resolution enhancement are two examples of inverse problems. The aim of this study is to develop novel and robust estimation approaches rooted in fundamental statistical concepts that could be utilized in solving several inverse problems in image processing and potentially in image reconstruction. In this study, we have implemented Bayesian methods that have been identified to be particularly useful when there is only limited data but a large number of unknowns. Specifically, we implemented a locally adaptive Markov chain Monte Carlo algorithm and analyzed its robustness by varying its parameters and exposing it to different experimental setups. As an application area, we selected radionuclide imaging using a prototype gamma camera. The results using simulated data compare estimates using the proposed method over the current non-locally adaptive approach in terms of edge recovery, uncertainty, and bias. The locally adaptive Markov chain Monte Carlo algorithm is more flexible, which allows better edge recovery while reducing estimation uncertainty and bias. This results in more robust and reliable outputs for medical imaging applications, leading to improved interpretation and quantification. We have shown that the use of locally adaptive smoothing improves estimation accuracy compared to the homogeneous Bayesian model.

## 1 Introduction

As a non-invasive method, medical imaging is extensively used for diagnosing and monitoring various medical conditions (1). However, the loss of information during the scanning and image acquisition processes often creates an observed image that is blurred and contains noise (2). The systematic relationship between the observed and true image is often modeled linearly using a transformation matrix. However, this transformation matrix is typically large and ill-posed, so directly solving a system of linear equations to obtain the exact image is infeasible.

In medical image processing, Bayesian modeling transforms an ill-posed problem into a well-posed problem by introducing a prior distribution as a form of penalization or regularization. Moreover, this method holds potential for application in biomedical image reconstruction (3, 4). Most approaches, however, have the tendency to not only smooth out noise but also to smooth out the signal. This raises the question of how to determine a prior distribution for smoothness in order to avoid both under- and over-smoothing. Homogeneous prior distributions have been found to be less effective in scenarios with rapid changes, such as medical images (5, 6). Inhomogeneous Bayesian modeling aims to fully utilize the distribution’s properties. Instead of employing different prior distributions, one could consider using a prior distribution with hyper-prior parameters (7). Therefore, we integrate inhomogeneous factors into the modeling by updating our prior distribution, introducing locally adaptive hyper-prior parameters, with high dimensions, instead of a single global hyper-parameter.

## 2 Materials

Given the absence of real images in practical scenarios, certain statistical measurements such as mean squared error (MSE) evaluating the efficiency of statistical modeling by minimizing the difference between real and estimated values are limited in application. Hence, creating simulated data to mimic the real image is required to bridge this gap.

### 2.1 Designed simulation with high contrast

The process for creating simulated data is as follows: we aim to generate simulated data X that closely approaches the true image represented as “real data.” We then apply random noise to create the degraded observed data Y. In this case, instead of using the projection data P in the posterior, we can obtain estimations by sampling from the posterior conditional distribution given Y. This approach also allows for a comparison between estimations and the corresponding real data X. The simulation is based on the function minimum residual sum of squares (RSS), where we can adjust the parameters to achieve simulations with different levels of noise. The general expression of the function is

$$\min_{\theta }{\left\|Y-A\left(\delta \right)X\left(r,c,z,T\right)-\epsilon \right\|}^{2},$$

where r is the collection of four objects’ radius: $r=(r_{1},r_{2},r_{3},r_{4}{)}^{T}$; $c=(c_{1},c_{2},c_{3},c_{4}{)}^{T}$ and $z=(z_{1},z_{2},z_{3},z_{4}{)}^{T}$ represent the parameter vectors of the objects’ central position of *x*-axis and *y*-axis, respectively. ϵ represents random errors introduced during observation. Given the prior information, the density for each cylinder is identical, denoted as T.

The elements within A represent the probabilities that pixels in X can be transformed into corresponding information in Y. In our application, $a_{ij}$ follows a bivariate-normal distribution with zero covariance:

$$a_{ij}∼N\left(\mu ,\Sigma \right),$$

where μ=(0,0) and $\Sigma =\delta^{2}\;I_{2\times 2}$ (I is an identity matrix). The standard deviation δ is highly dependent on the distance between the scanner and the scanner object. The transformed information from X to Y decreases with increasing distance. Ideally, when there is no distance between the scanner and the object, A is an identity matrix, allowing for complete information transformation. In medical imaging, despite the scanner’s position being fixed during an examination, the distance may vary slightly depending on the size of the object. Hence, when there is a considerable distance between the scanner and scanned objects, information is missing due to the reception of limited signals. Conversely, increasing the distance introduces more blurring. Overall, it is crucial to strike a balance between maintaining information and reducing blurring simultaneously; in other words, deciding how to set δ becomes vital.

### 2.2 Designed simulation with high contrast

To generate simulations, we set values for parameters. Afterward, random noise is applied to create the degraded observation Y. In this case, we can obtain estimations by sampling from the posterior conditional distribution given X (8). This approach also allows for a comparison between estimations and the corresponding real data X. The simulations are stored in pixel matrices of the size 29×58.

**Figure 1:**
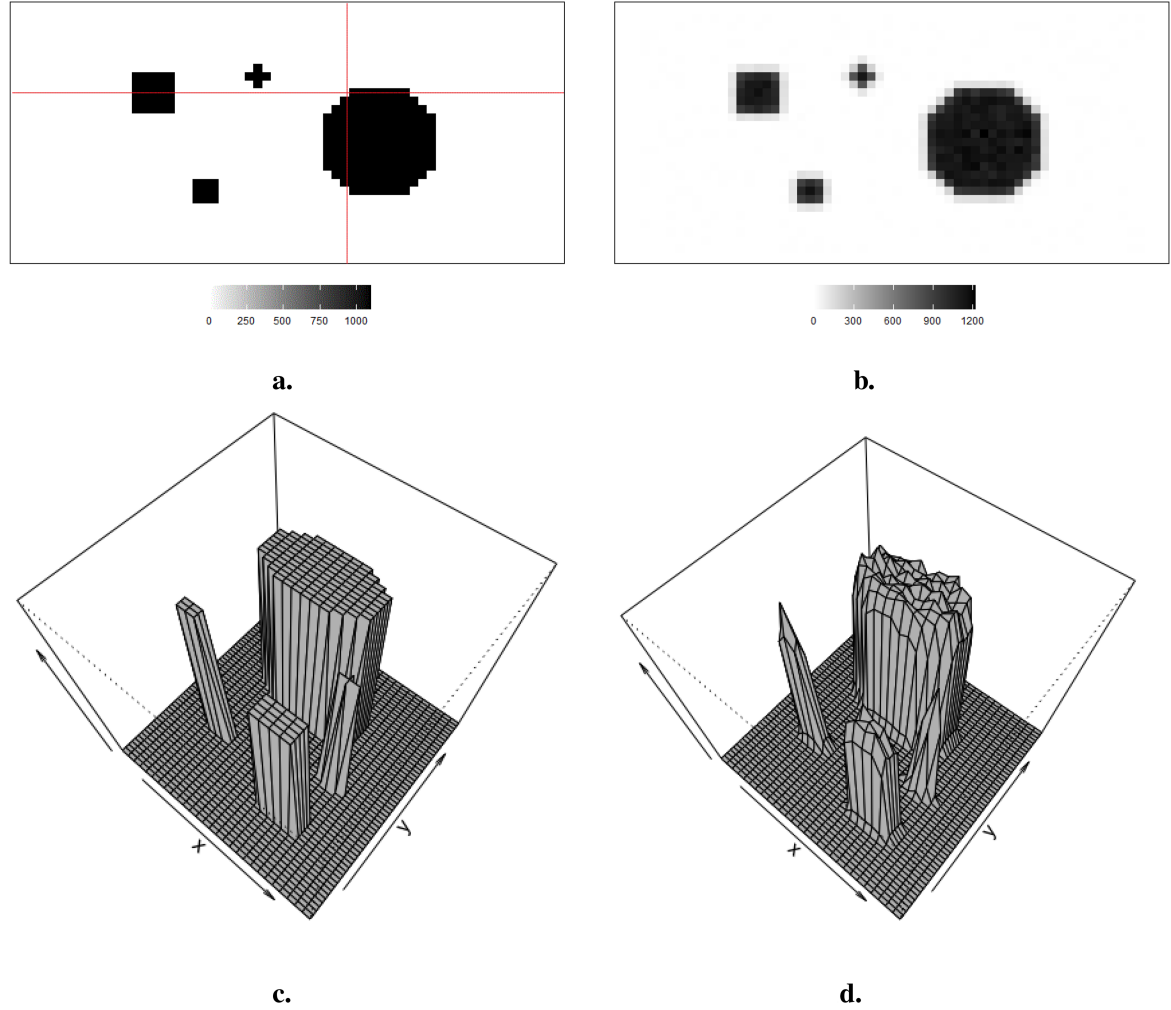
Simulation datasets: true information X and its correspondingly observation data Y. **(a)** Simulated image X. **(b)** Observation Y. **(c)** 3D simulated image X. **(d)** 3D observation Y.

Observed data Y, viewed as a degraded version of the actual distributions X with blur and noise, is comparable to the projection dataset in reality. As shown in Figure 1a, the true distribution consists of four sharp regions with sharp boundaries. The red lines indicate the regions of interest, namely, the 20th row and the 36th column, which are applied in the following pixel estimations. Figure 1b depicts observed data with low contrast resolution; blur is evident around the edge of each region.

**Figure 2:**
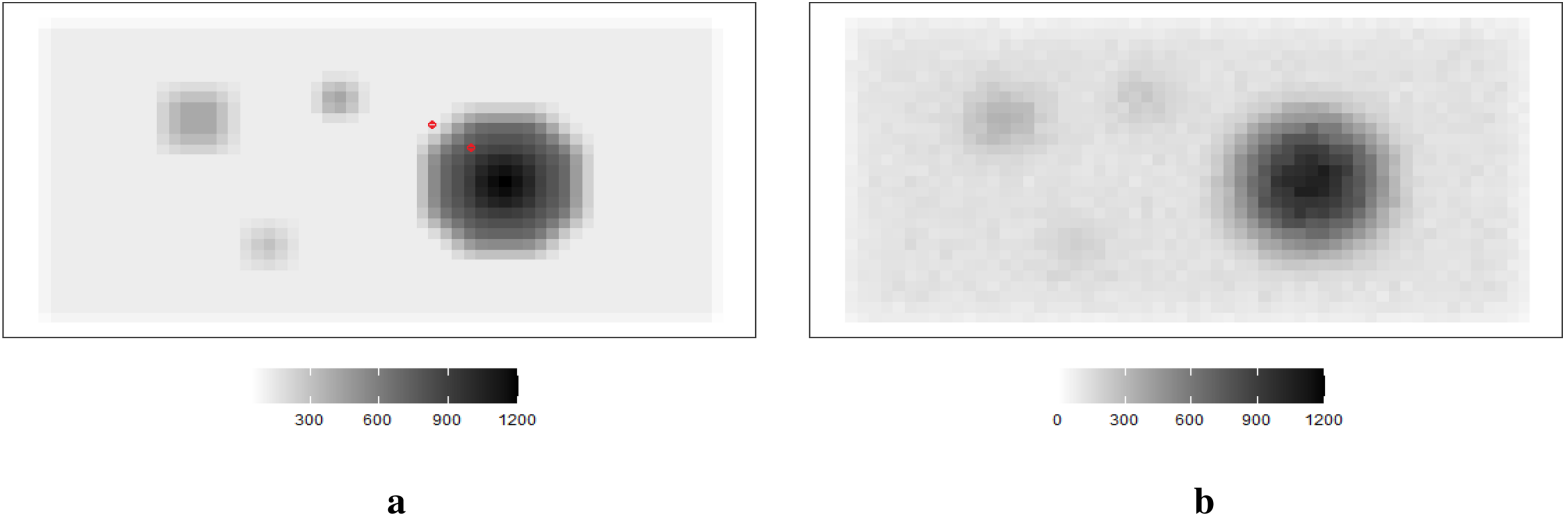
Simulation datasets with a reduction in scanning time: true image $X_{1}$ (left) and observed image $Y_{1}$ (right). **(a)** Simulated image $X_{1}$. **(b)** Observation $Y_{1}$.

**Figure 3:**
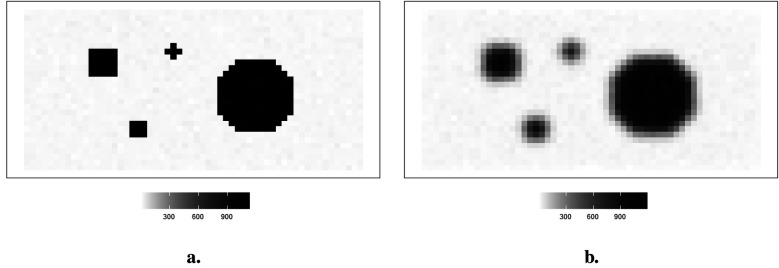
Simulation datasets with scanning time reduction: observed image $Y_{2}$ and its corresponding actual image X. **(a)** Simulated image X. **(b)** Observation image $Y_{2}$.

The hot region and background pixels are constant, around 1,100 and 0, respectively. In the three simulations, the noise presents in different ways. The first simulated dataset has the lowest noise compared to the other two datasets since there is a distinguished value gap between the background, where the pixels are close to zero, and the hot region, where the pixels are close to 1,100. In the second simulated dataset in Figure 2, it creates an environment where the hot regions are soft tissues with smooth edges; the average value of pixels in the background is higher than in other experiences, while the peak values are smaller than others. The red dots indicate the positions of pixels applied in the following pixel estimation comparison. Finally, the ones in Figure 3 describe the situation when there is a reduction in scanning time. There is a higher noise level than the first simulation. These simulations with artificial noise are regarded as degraded images. The proposed methods are assessed using simulated examples designed specifically to mimic real experimental data collected as part of system calibration experiments.

### 2.3 Designed simulation with smoothing edge

Supposing the objects within the circle have a soft edge instead of a hard one. In reality, the edges are likely to be considerably softer. Therefore, a smoothing pattern is obtained by applying a Gaussian kernel filter to the datasets presented in Figure 2a. If G is a Gaussian kernel, we say that $X_{1}=G\times X$. The high blurring around the high-contrast edge between the hot regions and the background makes it difficult to detect the original edge.

### 2.4 Simulated experiment with lower detected counts

Image quality improves with a reasonable extension of scanning duration. Again, supposing the scanning time is reduced, the observation image contains blurring. Another degraded simulated image is created, as shown in Figure 3. The high-contrast edge between the hot regions and the background blurring and the observation image is expected to contain more noise than the first observation image Y.

Ultimately, these simulated Y, $Y_{1}$, and $Y_{2}$ serve as our “observed dataset” for the application sections in this paper, while the simulated X remains an unknown parameter that needs to be estimated. However, as the values of simulated X are obtained in advance, it allows for a comparison between simulated X and its estimations from our defined posterior distribution in the following step.

## 3 Methods

In the case of medical imaging, the aim is to estimate a discrete version of the unknown continuous emitter activity distribution X from the single projection data Y. Suppose the unknown object X is expressed as a set of m volume pixels, $X=(X_{j}\;:\;j=1,2\ldots ,m)$, where $X_{j}$ represents the constant value of emitter activity in the jth pixel. The data $Y=(Y_{i}\;:\;i=1,2\ldots ,n)$ is related to the *actual activity* through the deterministic equation E(Y)=f(X) and depending on the application being studied, f(X) can become a linear function, or remain a non-linear function, especially when scanning time and multilayer factors are considered (9–11).

### 3.1 Likelihood function

A Poisson form, identified as suitable for various image processing with quantum noise, is particularly appropriate for the γ-${\text{eye}}^{\text{TM}}$ camera projection data considered in our application (12–14). The first γ-${\text{eye}}^{\text{TM}}$ scintillation camera developed by BIOEMTECH (Athens, Greece) was used to generate two-dimensional medical images (15).

The conditional distribution for observation Y given the unknown true radionuclide distribution X is as follows:

(1) $$f_{Y|X}\left(y_{1},y_{2},\ldots ,y_{n}|x\right)=\prod_{i=1}^{n}\lambda_{i}^{y_{i}}\frac{\exp\left(-\lambda_{i}\right)}{y_{i}!},$$

where $E[Y_{i}]=\lambda_{i}=\sum_{\;j=1}^{m}a_{ij}x_{j}$, j=1,2,…,m. In other words, each projection data value has an interaction with the whole vector X; a known transformation matrix is denoted $A=[a_{ij}{]}_{n\times m}$. The element $a_{ij}$ is the probability that a gamma particle emitted from pixel location i is recorded at pixel location j. The error ϵ can be expressed as an n×1 vector with elements $\left(\epsilon_{i}\;:\;i=1,2,\ldots ,n\right)$, which may come from various types of unavoidable measurement errors.

If the transformation matrix A is square and non-singular, image X can be easily realized by the least squares estimator $X\leftarrow \arg\min_{X}\;\|Y-AX{\|}^{2}$, when the squared error function is minimized:

$$\hat{X}={\left(A^{T}\;A\right)}^{-1}\;A^{T}\;Y,$$

where $\hat{X}$ is the least square estimate of X. However, in image processing, the transformation matrix A typically has a complex structure with high dimensions and is rectangular with unavailable pseudo-inverse. This results in ill-posed and ill-conditioned issues (16).

By imposing additional constraints in terms of prior knowledge, a Bayesian approach transforms an ill-posed inverse problem into one that is well-posed (17). During the modeling process, a prior distribution is constructed to capture the statistical properties of the image, and then estimation uses a posterior distribution derived by the combination of prior and likelihood. The uncertainty between X and Y is captured by likelihood function $f_{Y|X}(y|x)$ and the posterior density $f_{X|Y}(x|y)$ is used for inference after incorporating prior knowledge $\pi_{X}(x)$.

### 3.2 Prior distribution

Discrete images comprise elements of finite product spaces, and the probability distributions on such sets of images as prior information are a critical part of image processing. The efficiency of prior distributions depends on the available first-hand information. Regarding informative priors, it is generally expressed as the Gibbs measure, which was borrowed from statistical physics (18). The primary goal of introducing this type of probability is to describe the features relative to “macroscopic” physical samples, such as an “infinite system” (19, 20).

The Gibbs probability distribution has gradually found applications in various fields, including “Gibbs Sampling” in Bayesian modeling. The Gibbs distribution is defined as

(2) $$\pi_{X}\left(x|B\right)=Z^{-1}\exp\left(-B\kappa \left(x\right)\right),\;Z=\int_{x}\exp\left(-B\kappa \left(x\right)\right)dx,\;X\in R^{m},B>0,$$

where Z is the normalization for the Gibbs distribution; the energy function is κ, representing the energy of the configuration of pixels; and B is a non-negative smoothing parameter. Furthermore, the energy function can be rewritten as the sum of local energy functions Φ(⋅):

(3) $$\kappa \left(x\right)=\sum_{\;j=1}^{m}\Phi_{j}\left(x\right),$$

where $\Phi_{\;j}(\cdot )$ represents the local energy function to corresponding $X_{j}=x_{j}$.

#### 3.2.1 Markov random field for pixel differences

**Figure 4:**
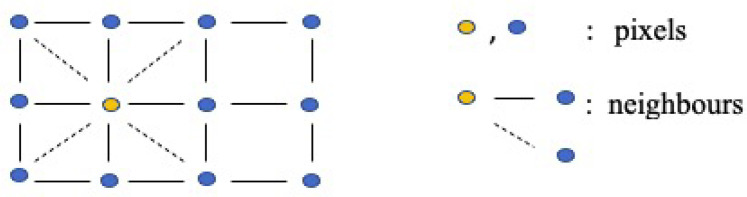
Two-dimensional rectangular grid G. The blue and yellow nodes in the lattice represent pixels, while the solid lines and dashed lines describe first-order and second-order neighborhoods, respectively.

Briefly, the primary assumption for Markov random field (MRF) models is that a variable is only related to its adjacent variables while being conditionally independent of the others (21, 22). Specifically, the clique-based structure makes MRF models well-suited for capturing local pixel relations in images. It proposes a lattice system, denoted as G=(V,E), to represent the connections between pixels (as illustrated in Figure 4). For instance, assuming the yellow node represents the object under analysis in the first-order system, its four closest neighbors are located to its left, right, bottom, and top sides, as indicated by the black solid lines. In the second-order system, an additional four neighborhoods are considered, located at the top-left, top-right, bottom-left, and bottom-right corners around the yellow node, as indicated by the dashed lines. In this system, pixels are represented as nodes (V), and edges (E) connect all the nodes. While the shape of the grid is not required to be rectangular, it is the most common in applications.

Once we define the MRF prior, the local function in Equations 2, 3 is rewrite a linear combination of differences between the pixel and its neighborhoods:

(4) $$\Phi_{\;j}\left(x\right)=\sum_{t\in \partial \left(\;j\right)}w_{\;jt}\phi \left(x_{j}-x_{t}\right),$$

where $w_{\;jt}$ represents the weight for each paired comparison, as the increased order in MRF, and $w_{\;jt}$ may change according to the interaction within the neighborhood. For example, for the first-order MRF, $w_{\;jt}=\;0.5$ if there is only four neighbors to consider. The set of nodes ∂(j) in a finite graph X with edges j∼t. Finally, after employing the Markov random field for pixel difference, the updated prior distribution is

(5) $$\pi_{X}\left(x|B\right)=Z^{-1}\exp\left(-B\sum_{\;j=1}^{m}\sum_{t\in N_{\partial \left(\;j\right)}}\;w_{\;jt}\phi \left(x_{j}-x_{t}\right)\right).$$

Here, only local characteristics are considered when it comes to estimating the individual pixel, which can be briefly divided into two properties. First, in comparison to the global character which includes all the pixels, we only study the smaller number of pixel’s neighbors; for instance, the first-order neighborhood is adopted in the prior distribution. Second, given the local property of the prior distribution, we assume that the posterior is sensitive to the local property of the prior distribution. If we define the potential functions as absolute value function, then ϕ(μ)=|μ| respectively. Thereby, the corresponding priors are quantified Markov random fields with absolute function (LMRF) combined with Equations 4, 5:

(6) $$\pi_{X|\tau }\left(x|\tau \right)=\prod_{\;j=1}^{m}\frac{1}{\left(2\tau_{j}\right)}\exp\left(-\frac{\sum_{t\in \partial \left(\;j\right)}|x_{j}-x_{t}|}{\tau_{j}}\right),\;x_{j}\geq 0,\tau_{j}>0,$$

where $X=\{x_{j},j=1,2,\ldots ,m\}$ and $x_{j}$ is conditional based on the neighbor’s values and $\tau =\{\tau_{j},j=1,2,\ldots ,m\}$ is the local conditional variance in the prior distribution, which accounts for the value variances among individual pixels and its four neighbors. As a comparison to Equation 6, we define a homogeneous prior variance τ to capture the global variance between the pixel differences:

$$\pi_{X|\tau }\left(x|\tau \right)=\prod_{\;j=1}^{m}\frac{1}{\left(2\tau \right)}\exp\left(-\frac{\sum_{t\in \partial \left(\;j\right)}\;|x_{j}-x_{t}|}{\tau }\right),\;x_{j}\geq 0,\tau >0.$$

The inhomogeneous prior relies on the contrast levels in the image’s segmentation, making it locally adaptive to various image densities. In order to cover all scenarios among pixel neighborhoods, the modeling with inhomogeneous hyper-parameter τ can tolerate a large value fluctuation and detect a small variation within the smoothing areas.

#### 3.2.2 High-dimensional Markov random field

**Figure 5:**
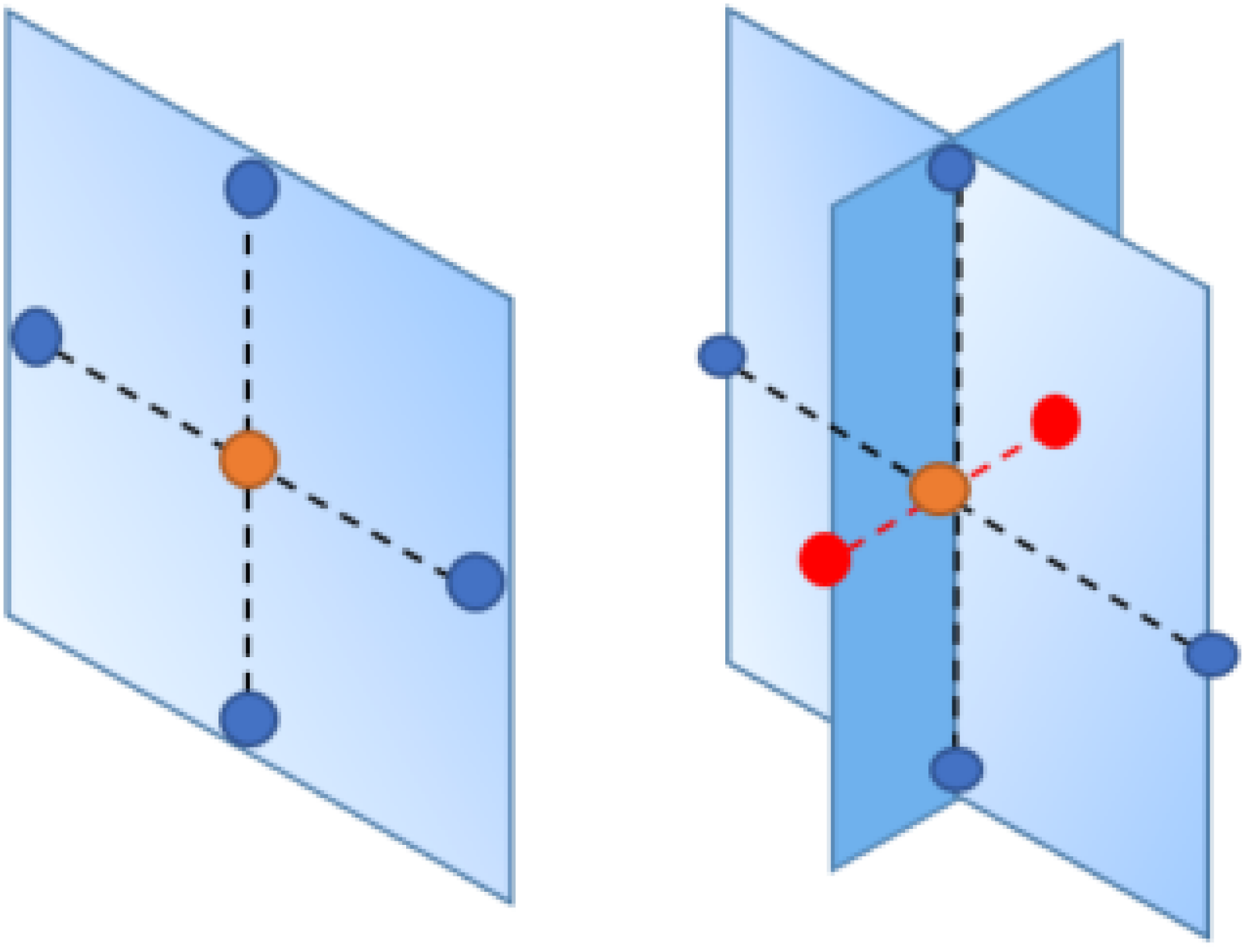
Markov random field in three-dimensional space. Left image: Markov random field in two-dimensional space. Right image: Markov random field in three-dimensional space. The nodes with different colors in the lattice represent pixels, while the dashed lines describe the connection between the objective pixel (orange node) and its neighbors.

The image dataset presents a two-dimensional image in our application. Hence, we only consider the first-order system in Markov random field priors, where the pixel and its four closest neighbors are on the same planet. However, if the application dataset from the projection data to the tomography image, where pixels within two-dimensional space transfer into voxels within three-dimensional space, we can introduce another two neighbors of pixels based on the first-order system. As seen in Figure 5, the left side, as a comparison to the right side, displays the Markov random field within two-dimensional space. There are two additional red nodes applied based on the first-order system.

The Markov random field prior distribution can still be written as a general form of Gibbs distribution:

$$\pi_{X}\left(x|B\right)=kB^{m}\exp\left(-B\sum_{\;j=1}^{m}\sum_{t\in N_{\partial \left(\;j\right)}}\;w_{\;jt}\phi \left(x_{j}-x_{t}\right)\right),\;x_{j}\geq 0,\tau >0,$$

where the number of neighbors in $N_{\partial (j)}$ increases from four to six neighbors in three-dimensional space.

#### 3.2.3 Hyper-prior distribution

In the inhomogeneous model, τ is a vector of unknown local parameters which can either be defined by allocating a series of artificial values or by introducing another level of modeling that correctly incorporates the additional uncertainty. The first solution requires advanced information for the assignment which is not usually available. Alternatively, the second action employs a hyper-prior distribution, $\pi_{\tau }(\tau )$, which is diffuse unless there is more informative information available. Here, an exponential distribution is used for each $\tau_{j}$ independently but with a common rate parameter, γ, that is, $\tau_{j}|\gamma \;∼\;\text{Exp}(\gamma )$. The value of γ is chosen to produce a long-tailed distribution to cover varied scenarios. As well as promoting smaller values of $\tau_{j}$ compared to a non-informative uniform distribution, it also avoids the need to impose an arbitrary upper range limit. The distribution for $\pi_{\tau |\gamma }(\tau |\gamma )$:

(7) $$\pi_{\tau |\gamma }\left(\tau |\gamma \right)=\prod_{\;j=1}^{m}\gamma \;\exp\left(-\gamma \;\tau_{j}\right),\;\gamma >0.$$

The elements in $\tau =\{\tau_{\;j};\;j=1,2,\ldots ,m\}$ are the collection of local prior variances and γ refers to the rate parameter in the hyper-prior distribution, a smaller value for the rate parameter indicates the more flat hyper-prior distribution.

#### 3.2.4 Rate parameter in the hyper-prior distribution

**Figure 6:**
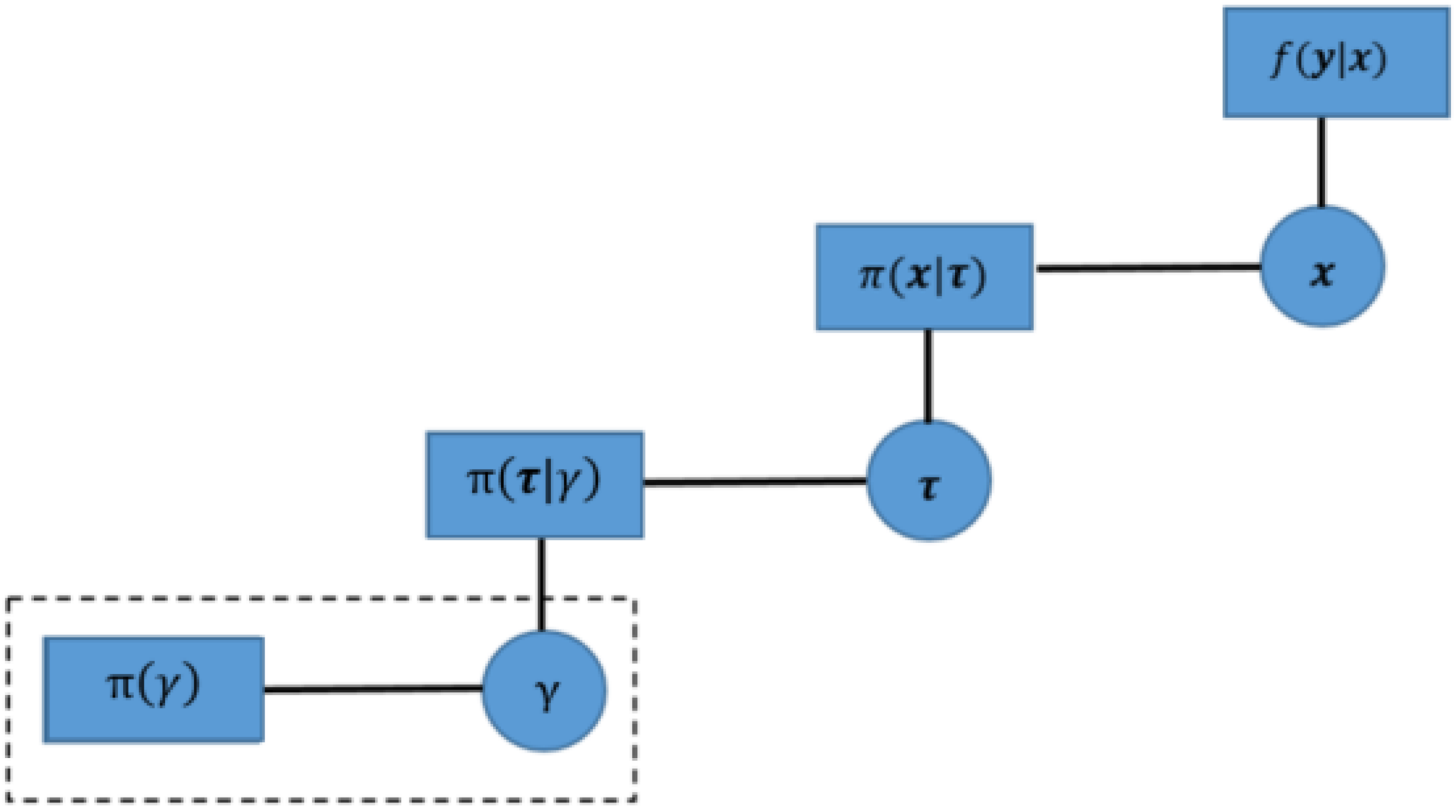
Factor graph for hierarchical Bayesian model.

The fundamental strategy for establishing a hierarchical Bayesian multilevel model is specifying prior distributions for each unknown parameter, which enables estimation for each parameter based on the other prior distributions from different levels. Therefore, it can incorporate more prior knowledge and hence improve estimation accuracy. However, once additional prior levels are involved in the model, it can result in a prolonged computation time and a high demand for supportive information. Figure 6 explains the multilevel structure in our hierarchical Bayesian model.

Now that an additional hyper-parameter, γ, has been introduced, further modeling must be considered. Due to a lack of supportive prior knowledge, it is common to utilize a uniform distribution or a Jacobian transformation of a uniform distribution. Another option is to introduce a conjugate prior distribution which here would be the Gamma distribution with shape and scale parameters.

For the first method, the uniform prior allocates the same probability to each value within its defined range; it is subjective but hard to estimate values when the value is extremely small (23, 24). However, overly complex models have a high risk of poor performance (25). The use of a Gamma distribution unavoidably expands the number of unknown parameters. We expect, however, that the density peak of the Gamma distribution to be a small value and therefore, an exponential distribution with a small value for the rate parameter, θ, should be adequate: *ρ* exp(*Θ*).

The result of these multilevel models, combining likelihood function in Equation 1, prior distribution in Equation 6, and hyper-prior distribution in Equation 7, produces the following posterior distribution:

(8) $$\begin{array}{rl} \;f_{X,\tau ,\gamma |Y} & \left(x,\tau ,\gamma |\;y\right)\\ & =f_{Y|X}\left(y|x\right)\;\pi_{X|\tau }\left(x|\tau \right)\;\pi_{\tau |\gamma }\left(\tau |\gamma \right)\pi_{\gamma }\left(\gamma \right)\\ & \propto \;\prod_{i=1}^{n}\lambda_{i}^{\;p_{i}}\frac{\exp\left(-\lambda_{i}\right)}{p_{i}!}\left[\prod_{\;j=1}^{m}\frac{1}{\left(2\tau_{j}\right)}\exp\left(-\frac{\sum_{t\in \partial \left(\;j\right)}\;|x_{j}-x_{t}|}{\tau_{j}}\right)\cdot \gamma \;\exp\left(-\gamma \;\tau_{\;j}\right)\right]\\ & \;\cdot \exp\left(-\theta \;\gamma \right), \end{array}$$

where $X=\{X_{j},j=1,2,\ldots ,m\}$ represents the unknown radionuclide distribution, $\tau =\{\tau_{j},j=1,2,\ldots ,m\}$ are the locally adaptive prior variance parameters, γ is the hyper-parameter modeling τ, and Y: $\left\{Y_{i},i=1,2,\ldots ,n\right\}$ is the observed data.

There is a concern about the potentially unreliable posterior estimations from the hierarchical model, especially for estimating small values. Hence, instead of estimating the hyper-prior parameter γ, we consider a calibration experiment where a series of values for γ, from $10^{-4}$ to $10^{3}$, are used to investigate the trend in the mean squared error under different γ. In other words, instead of bringing another level of hyper-prior distribution, γ is fixed, but based on the calibration experiments rather than requiring prior knowledge.

**Figure 7:**
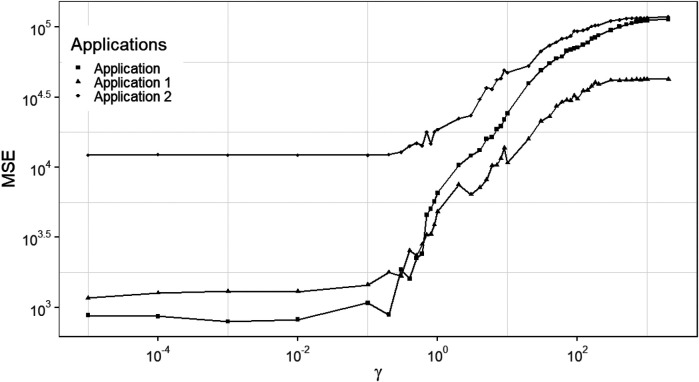
MSE of posterior estimation with fixed hyper prior parameter γ. Applications refer to the employment of simulation datasets (Y, $Y_{1}$, and $Y_{2}$), respectively.

As shown in Figure 7, a similar increased pattern of MSE occurs among the three data applications with different levels of noise and blurring. The applications are the three designed examples. The estimation using small γ, smaller than 0.1 say, is robust and based on mean squared error performance. Combining the posterior estimation for γ with the hierarchical Bayesian model, we can conclude that a rate parameter γ around $10^{-2}$ is a robust hyper-prior parameter value for our current application and hence in what follows $\gamma =10^{-2}$.

## 4 Results

### 4.1 Estimation strategy

Once the posterior distribution involving likelihood, prior, and hyper prior distribution is defined, as seen in Equation 8. A metropolis Hasting algorithm is used for the estimation. This is an example of the general Markov chain Monte Carlo (MCMC) approach that is able to handle complex distributions where other estimation methods fail. Details of the estimation process of our application can be found in Estimation algorithm of Markov chain Monte Carlo, Appendix Table A1. Apart from interest in the posterior estimation of the unknown radionuclide distribution, *X*, the locally-adaptive hyper-prior parameters *τ* must be estimated simultaneously. In general, the single global prior variance *τ* should capture the global variance between pixels. However, in the locally-adaptive model the hyper-prior variances *τ* measure each pixel's variation within the corresponding neighbourhood, and hence we expect the elements {***τ*** = *τ_j_*, *j* = 1, 2, . . . ,*m*} to be non-identical.

### 4.2 Posterior estimation

**Figure 8:**
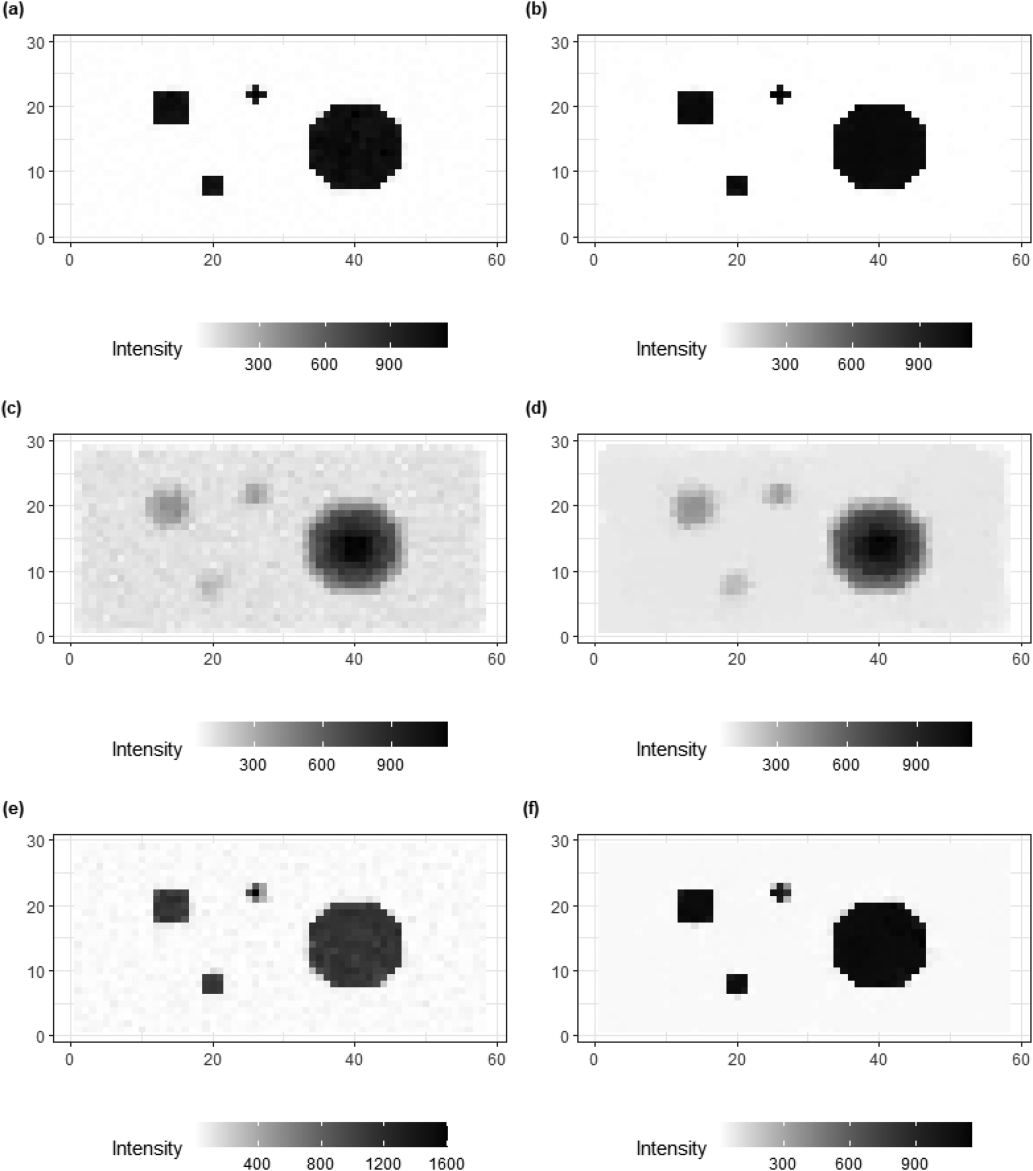
Comparisons of image processing for three simulations, X, $X_{1}$ and $X_{2}$, respectively. From the right side, **(a,c,e)** present the image processing with a global hyper-prior variance from the homogeneous Bayesian modeling. While **(b,d,f)** show the image processing under hierarchical Bayesian modeling with local hyper-prior variances.

Figure 8 shows the posterior estimates for the three scenarios, with X, $X_{1}$, and $X_{2}$. For comparison, posterior estimates using the homogeneous model (left) are shown along with the estimates using the inhomogeneous, locally adaptive prior model (right). After incorporating prior flexibility, estimation improvements are apparent for deblurring and denoising, particularly in the second and third applications of simulation datasets with high-level blurring.

In the first application, where more accurate and sufficient first-hand information Y is available, the results from both models (the homogeneous and inhomogeneous models, respectively) are similar. However, in the third application, despite the foundational truth being the same as in the first example, the first-hand information $Y_{2}$ contains high levels of noise and blurring. In this case, the hierarchical Bayesian modeling successfully produces an image based on $Y_{2}$ that is closer to the truth compared to the outcomes from the homogeneous modeling.

**Figure 9:**
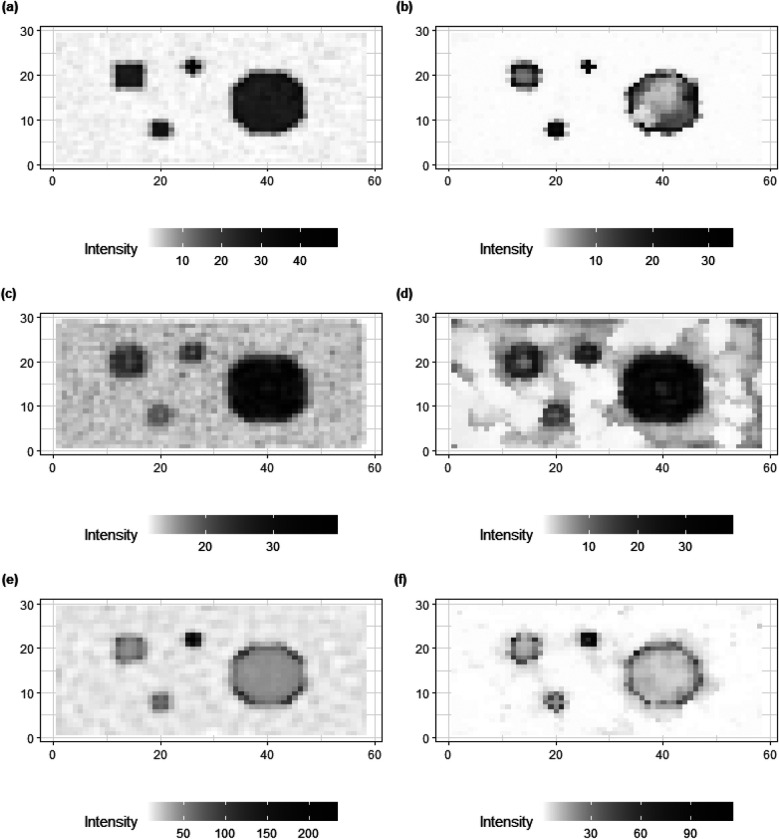
Standard deviation of posterior estimations for true X, $X_{1}$ and $X_{2}$ in image pattern. **(a,c,e)** present the image patterns of standard deviation that come from the homogeneous Bayesian model with a global hyper-prior variance. **(b,d,f)** show the image patterns of standard deviation originate from hierarchical Bayesian modeling with local hyper-prior variances.

**Figure 10:**
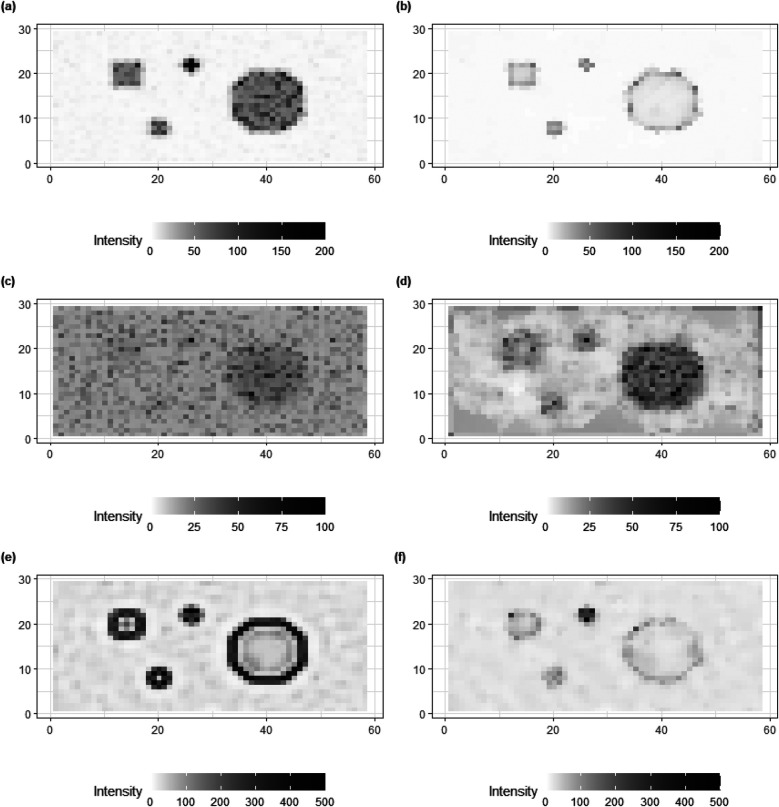
Bias of posterior estimations for truth X, $X_{1}$, and $X_{2}$ in image pattern. **(a,c,e)** present the image patterns of bias that come from the homogeneous Bayesian model with a global hyper-prior variance. **(b,d,f)** show the image patterns of standard deviation originating from hierarchical Bayesian modeling with local hyper-prior variances.

In addition to posterior estimates of X, standard deviation and bias values are shown in Figures 9, 10.

For standard deviation, the results from the homogeneous models (left) show higher values than the inhomogeneous models (right), especially for the hot regions and smoothing background. The variation can still be found in high-contrast edges while there is a reduction within the hot regions and smooth backgrounds where the pixel differences are assumed to be small.

The bias in Figure 10 provides clear additional evidence that the estimation accuracy of the locally adaptive model is improved compared to that of the homogeneous model. For instance, in Figure 10f, the bias around the high-contrast edge and smoothing hot regions is hardly detected in the right image as compared to the image on the left side. Generally, the image patterns of standard deviation and bias on the right side have less information observed compared to the left side. In other words, the adjusted model captures the variation and bias within the posterior estimations.

### 4.3 Comparisons of posterior pixel estimation

**Figure 11:**
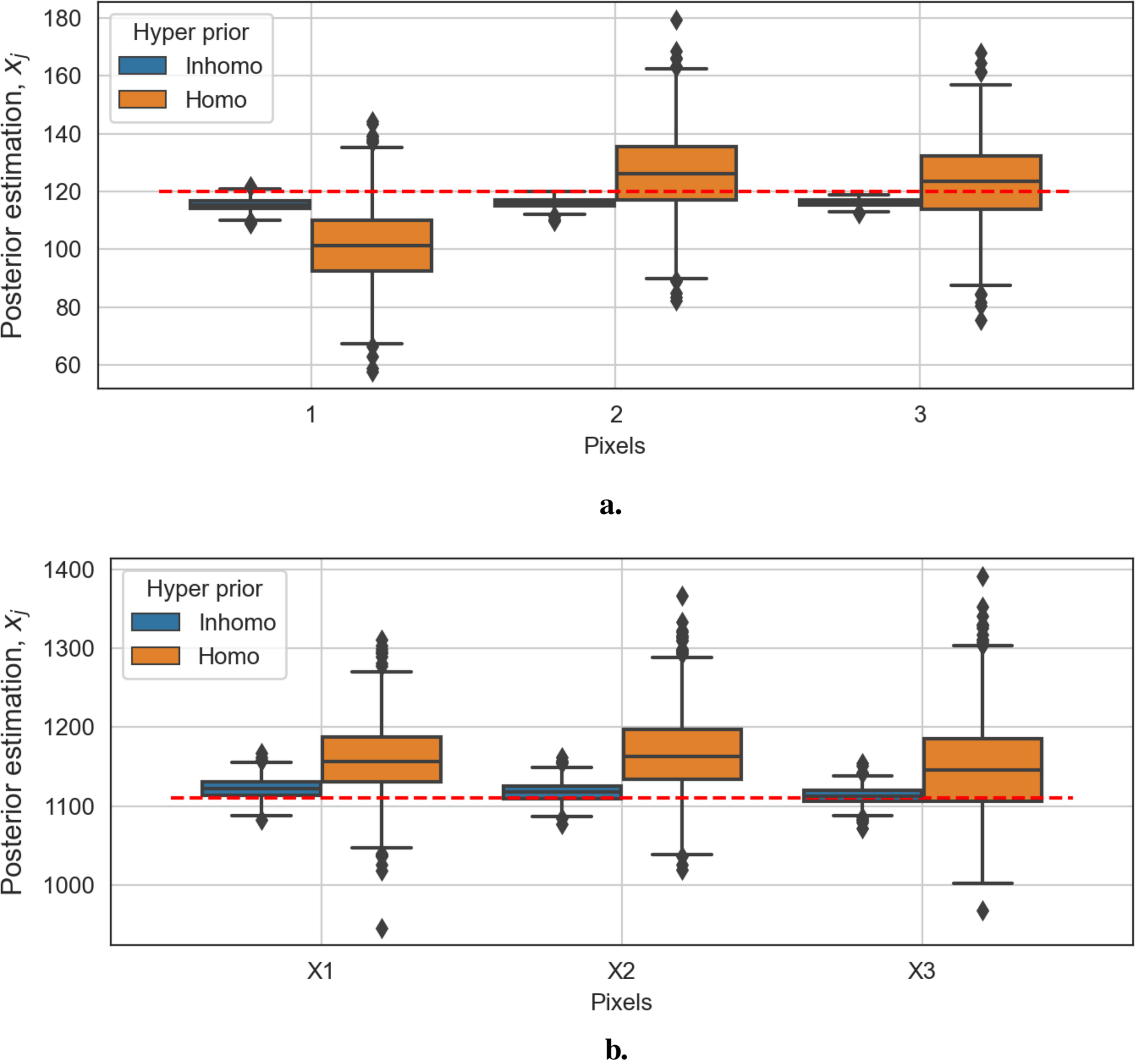
Box plots of posterior distributions. The box plots in blue show the results of the model with locally adaptive hyper-prior parameters, and the box plots in orange represent estimations from the homogeneous model. The red dashed line is the true pixel value. **(a)** Box plots of three background posterior distributions from the second example. **(b)** Box plots of three hot-region posterior distributions from the second example.

Figures 11a,b show box plots of three pixels from the background and three from the hot regions, respectively. The distribution in the blue box plots (left of each pair) is close to the red dashed line, representing the truth. In contrast, the ones in the orange box plots (right of each pair) have significant bias. Overall, the Bayesian model with locally adaptive hyper-prior variance introduces estimation flexibility to realize a more accurate outcome in each application.

**Figure 12:**
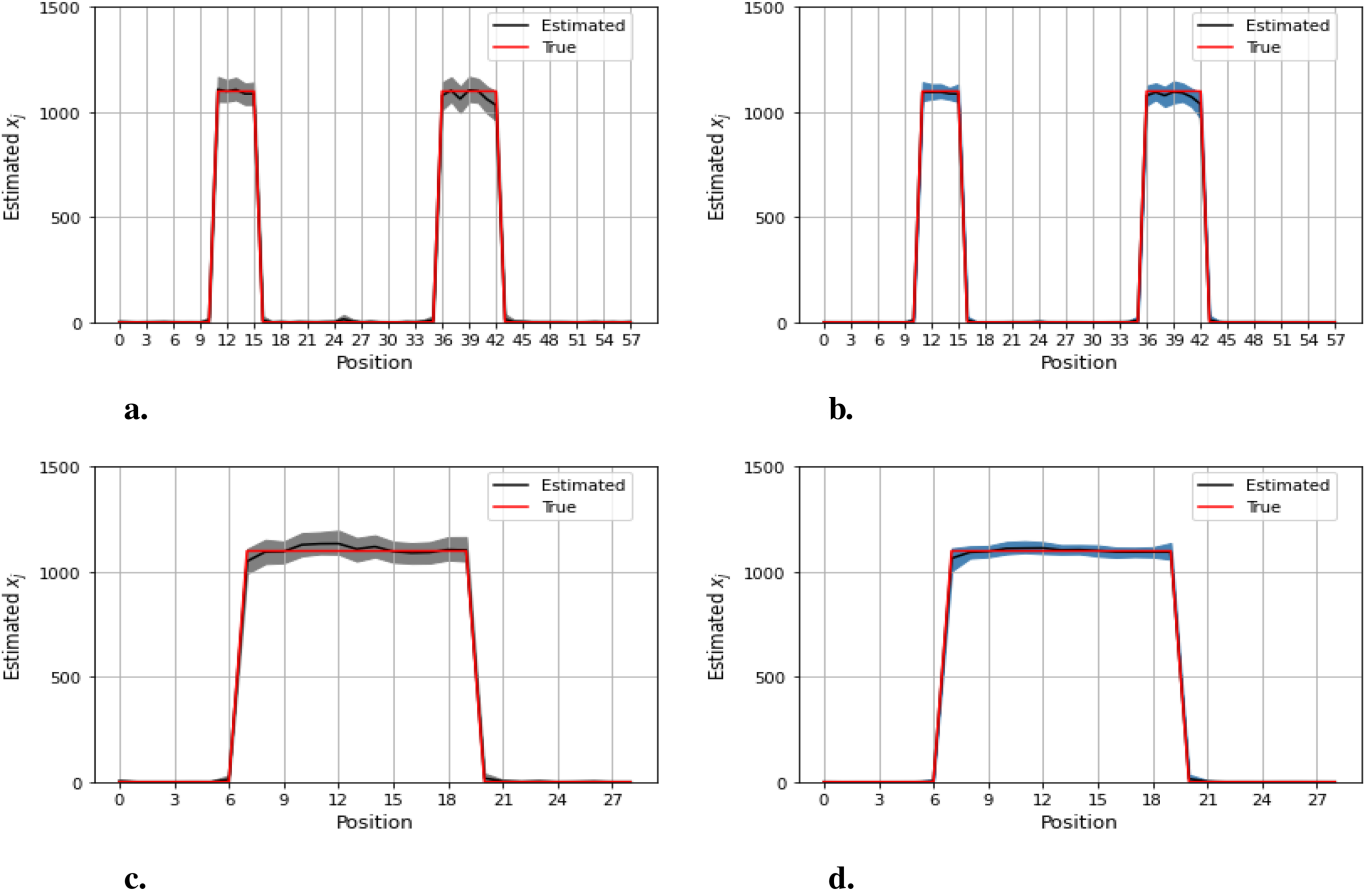
Pixel posterior distributions under homogeneous (left) and locally adaptive (right) models with hyper-prior variance parameters, using the first simulation dataset. The posterior distributions for the 20th row are shown at the top, while those for the 36th column are shown at the bottom. **(a,b)** Pixel estimates under the homogeneous and inhomogeneous modelling for the 20th row; **(c,d)** Pixel estimates under the homogeneous and inhomogeneous modelling for the 36th column.

**Figure 13:**
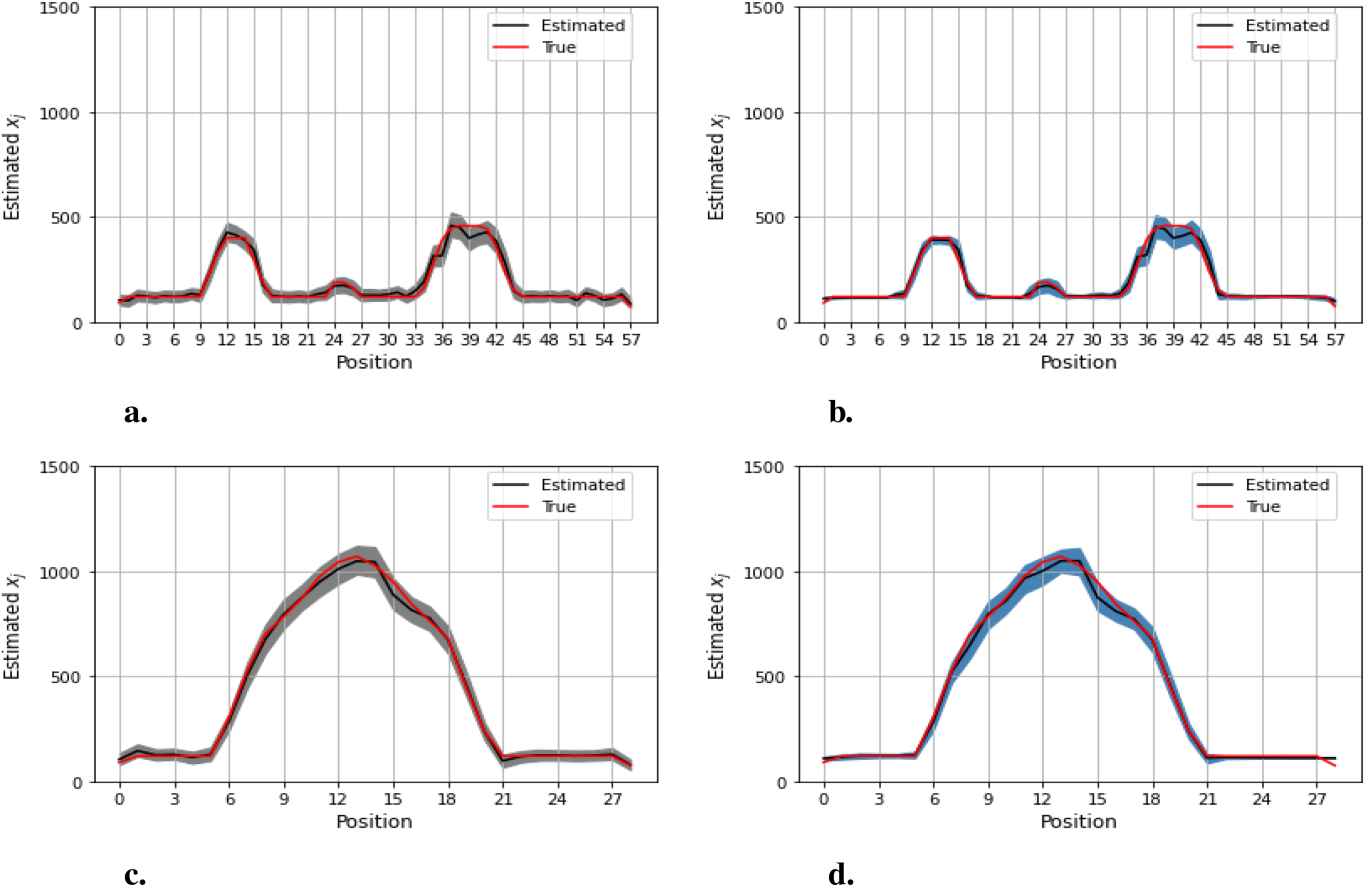
Pixel posterior distributions under homogeneous (left) and locally adaptive (right) models with hyper-prior variance parameters, employing the second simulation dataset. The posterior distributions for the 20th row are shown at the top, while those for the 36th column are shown at the bottom. **(a,b)** Pixel estimates under the homogeneous and inhomogeneous modelling for the 20th row; **(c,d)** Pixel estimates under the homogeneous and inhomogeneous modelling for the 36th column.

**Figure 14:**
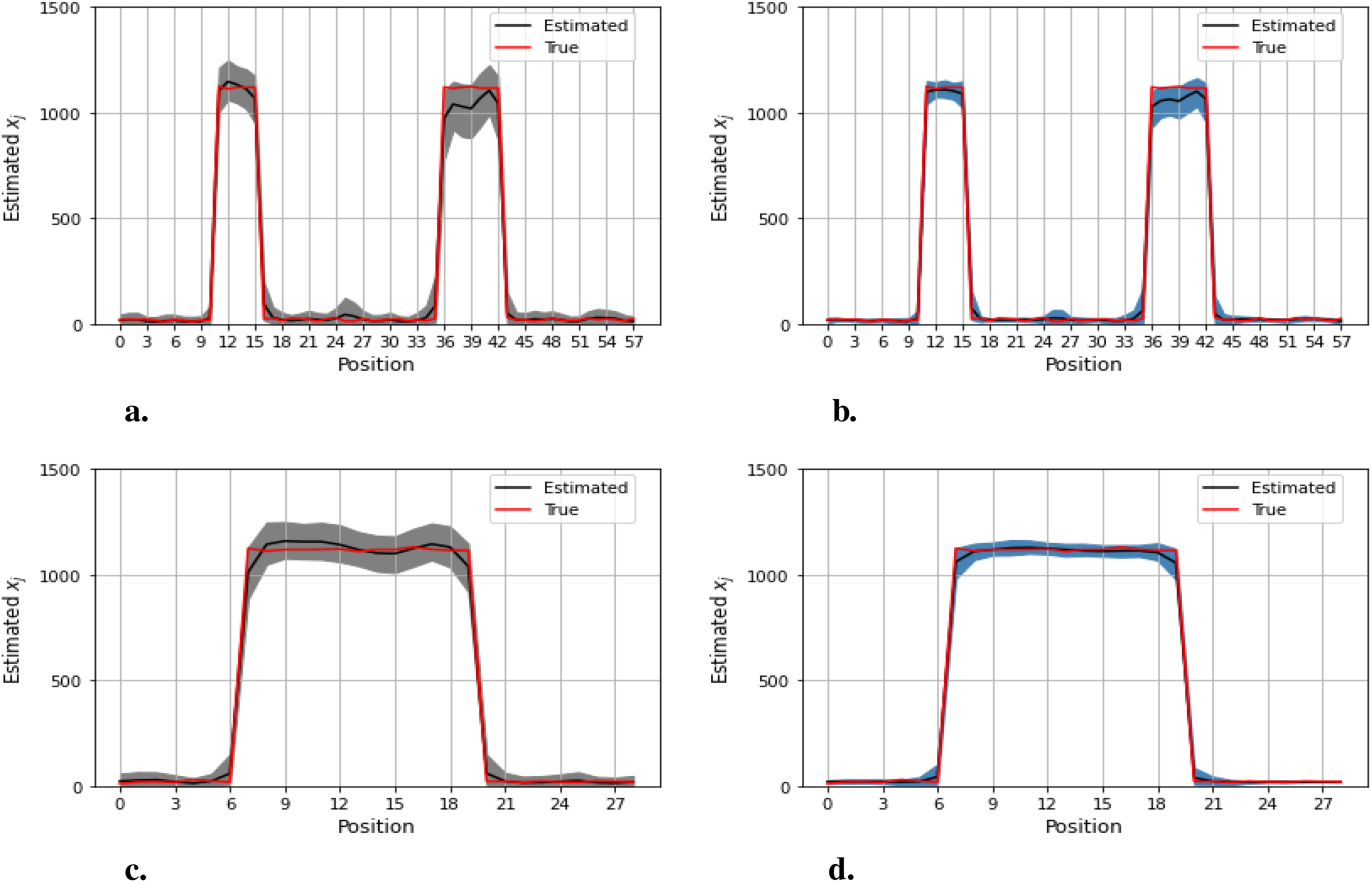
Pixel posterior distributions under homogeneous (left) and locally adaptive (right) models with hyper-prior variance parameters, employing the third simulation dataset. The posterior distributions for the 20th row are shown at the top, while those for the 36th column are shown at the bottom. **(a)**, **(b)** Pixel estimates under the homogeneous and inhomogeneous modelling for the 20th row; **(c)**, **(d)** Pixel estimates under the homogeneous and inhomogeneous modelling for the 36th column.

Figures 12–14 present comparisons of estimation pixels between the posterior containing homogeneous and inhomogeneous hyper-prior parameters in each simulation application (X, $X_{1}$, and $X_{2}$, respectively). In general, the estimations with a global hyper-parameter (left side) tend to have higher variations and broader credible intervals than those with locally adaptive hyper-parameters (right side), especially for the hot regions. Although the wider credible interval is more capable of covering variation in the values in comparison to the narrow credible interval, we noticed that there are some severe estimation fluctuations, especially within the smoothing pixel region, for example, in Figures 12, 13.

Furthermore, the value difference between the estimations from the locally adaptive model and the truth is smaller than the homogeneous ones. For instance, without sufficient first-hand information in the third simulation experience, as shown in Figure 14, the modeling with local hyper-prior variance produces a more accurate estimation as opposed to the one with global hyper-prior variance. Table 1 shows the corresponding estimation measurements of eight selected pixels in the 20th row in the third simulation application. Overall, the Bayesian model with locally adaptive hyper-prior variance introduces estimation flexibility to realize a more accurate outcome in each application.

**Table 1:** The list includes estimation measurements.

Position	ucl.H	ucl.In	lci.H	lci.In	mean.H	mean.In
1	45.36	20.60	0.90	18.47	15.80	19.58
4	32.58	20.09	0.48	16.81	11.46	18.67
8	35.69	23.06	0.59	13.86	12.18	18.36
14	1,219.05	1,136.55	1,045.48	1,086.63	1,132.85	1,111.66
18	79.04	30.05	2.13	15.27	29.53	21.53
22	62.27	24.30	1.66	16.50	23.93	20.46
24	48.84	25.52	0.55	16.01	16.94	20.55
26	123.85	43.67	3.40	9.73	44.13	24.86

“H” indicates the posterior estimation from Bayesian modeling with the global LMRF, while “In” indicates the posterior estimation from Bayesian modeling with locally adaptive LMRF. The outcomes are stored to two decimal places.

## 5 Modeling application in small animal imaging

**Figure 15:**
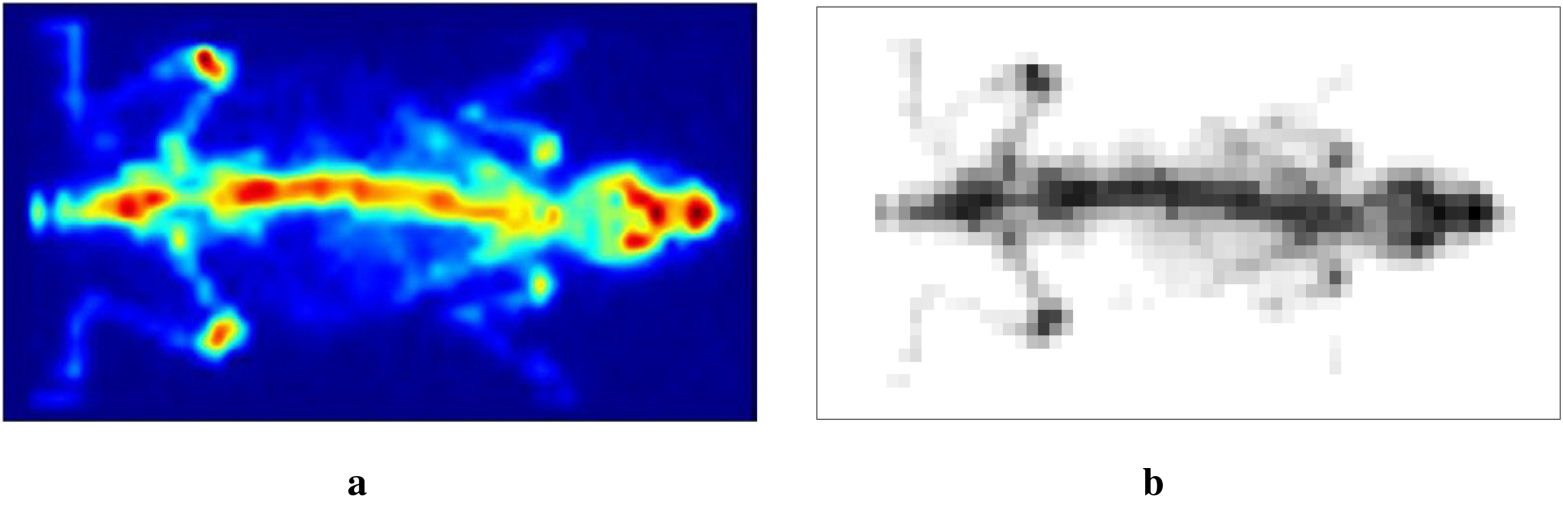
Scan of a mouse using γ-${\text{eye}}^{\text{TM}}$: **(a)** real scan and **(b)** a simulated dataset.

In the previous simulation examples, locally adaptive Bayesian modeling proves the advantages of estimation accuracy. We now aim to apply this technique to images obtained from mouse scanning by using γ-${\text{eye}}^{\text{TM}}$ to confirm the conclusion obtained from the former sections. Figure 15a shows the image of a mouse injected with a technetium-99m labeled radiotracer acquired with γ-${\text{eye}}^{\text{TM}}$, and Figure 15b presents the correspondingly designed dataset for estimation application.

**Figure 16:**
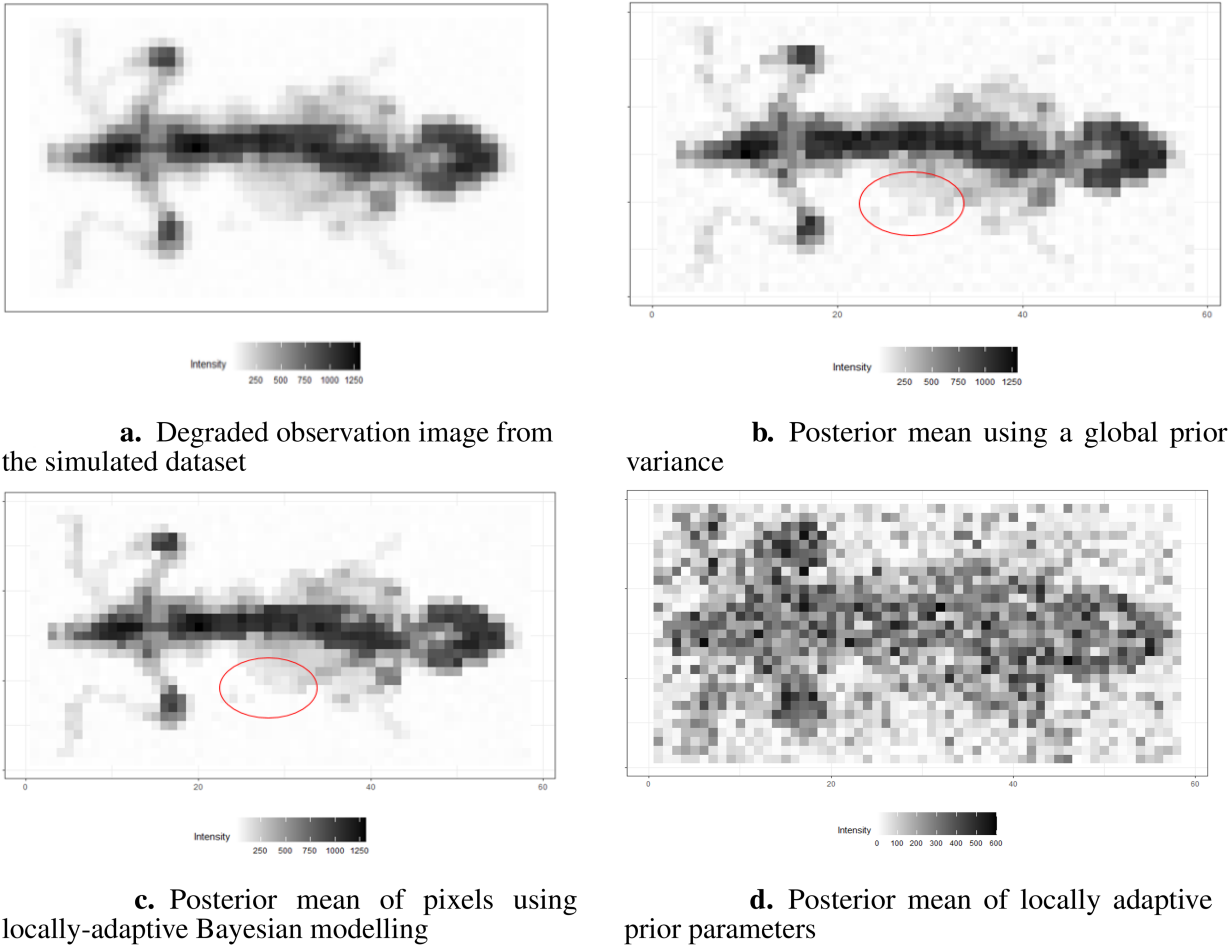
Image processing under the Bayesian modeling. **(a)** shows the simulation image with noise and blurring. **(b)** displays image processing under the Bayesian modeling with homogeneous hyper-prior parameter τ. **(c)** displays image processing under the Bayesian modeling with inhomogeneous hyper-prior parameter τ. **(d)** shows the posterior estimation of locally adaptive hyper-prior variances in the image pattern.

The results are presented in Figures 16b,c. Here, we assign the rate parameter $\gamma =10^{-2}$ in the hyper-prior distribution. As in the previous examples, the estimation with the locally adaptive hyper-prior variance performs better compared to the homogeneous model in terms of deblurring and denoising; for instance, the smoothing edge (red circle) between the background and hot region is clearer in Figure 16c than in Figure 16b. In Figure 16d, the estimation of hyper-prior τ is displayed, showing the clear non-identical value distribution of τ. The high-dimension τ introduces flexibility when estimating pixel variance.

The locally adaptive Bayesian model with inhomogeneous hyper-prior parameters can specifically describe the probabilistic distribution of each unknown pixel $x_{j}$. Beyond pixel-wise posterior estimation, these inhomogeneous hyper-prior variances enabled a more detailed exploration of the outcomes. For instance, plotting the estimated hyper-prior variances directly reveals spatial information about the pixels. In conclusion, the locally adaptive Bayesian modeling constructs a hierarchical network that encompasses multiple levels of parameters. This network effectively integrates information from estimated parameters across different levels.

## 6 Tikhonov regularization and real image application

The application of Bayesian modeling in cylinder simulation datasets demonstrates advantages in terms of deblurring and denoising. Therefore, to confirm the applicability of Bayesian modeling, another real-world data application is required. Tikhonov regularization has been identified as useful as it introduces the homogeneous regularization term into ill-conditioned problems, specifically in the context of inverse problems (26–28). Therefore, a comparison between the estimations from Bayesian modeling and Tikhonov regularization is necessary.

### 6.1 Tikhonov regularization comparison

Tikhonov regularization for medical image processing, which holds the linear relationship between observation image and real image, can be written as

$$\text{min}\left({\left\|Y-AX\right\|}_{2}^{2}+\lambda {\left\|LA\right\|}_{2}^{2}\right),$$

where A is the transformation matrix, and Y and X are the observation dataset and real unknown dataset, respectively. The regularization parameter λ controls the trade-off between the model fitness and the regularization term. The regularization matrix L contains the prior information about the solutions. Here, we employ the identity matrix as the regularization matrix L because of the lack of supportive prior information.

**Figure 17:**
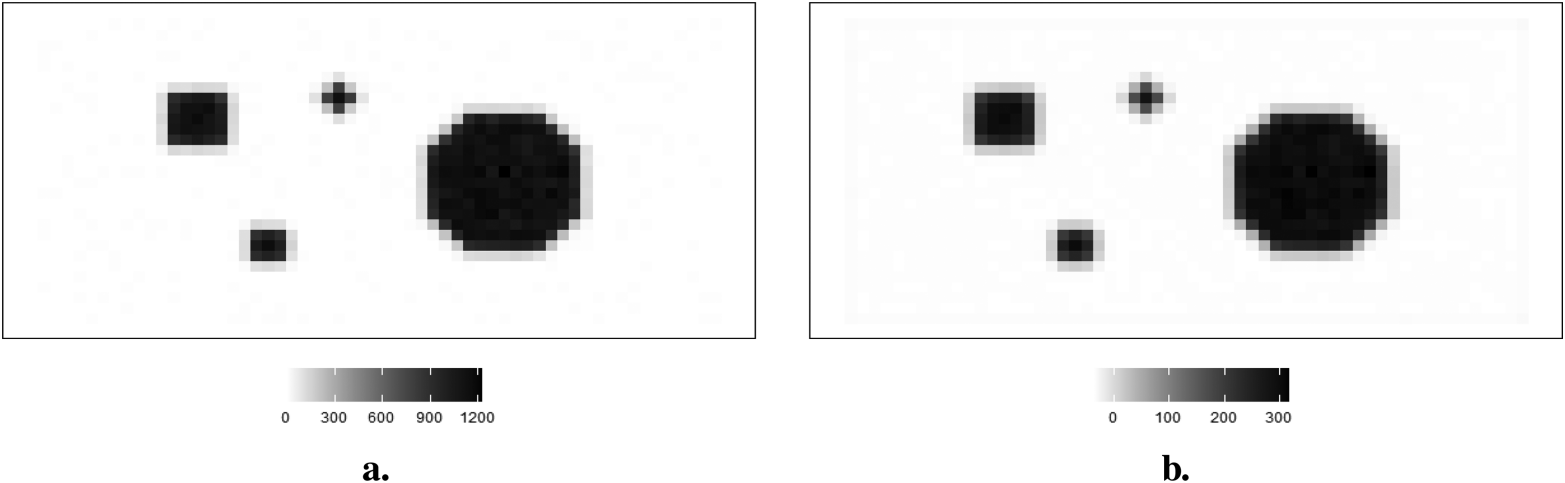
Observation image and the corresponding estimation from Tikhonov regularization with the optimum regulation. **(a)** Observation Y. **(b)** Tikhonov regularization.

In the context of the regularization parameter λ, the criterion of cross-validation has been applied in various regularization algorithms, including Tikhonov regularization. Cross-validation selects the optimum regularization parameter λ by identifying the minimum estimation residuals.^1^ The estimation outcome from Tikhonov regularization, using observation Y from within the first simulation dataset, is shown in Figure 17. The left side displays the observation of the first simulation dataset, while the right side presents the estimation from Tikhonov regularization.

Compared to the Bayesian application shown in Figure 8, the blurring in Figure 17b can still be detected around the high-contrast area between the background and hot region. Hence, the estimation for real image X is not accurate, since regularization applies to the whole information not only noise but also pixel values. In other words, the smoothing effect from regularization applies globally to pixels within both background and hot regions simultaneously, regardless of varied pixel densities. The estimated pixels with a large value of regularization (represented in green) are smoother than those with small regularization (represented in blue). Furthermore, some non-negative pixels from the background are unavoidably transformed into negatives after applying regularization.

**Figure 18:**
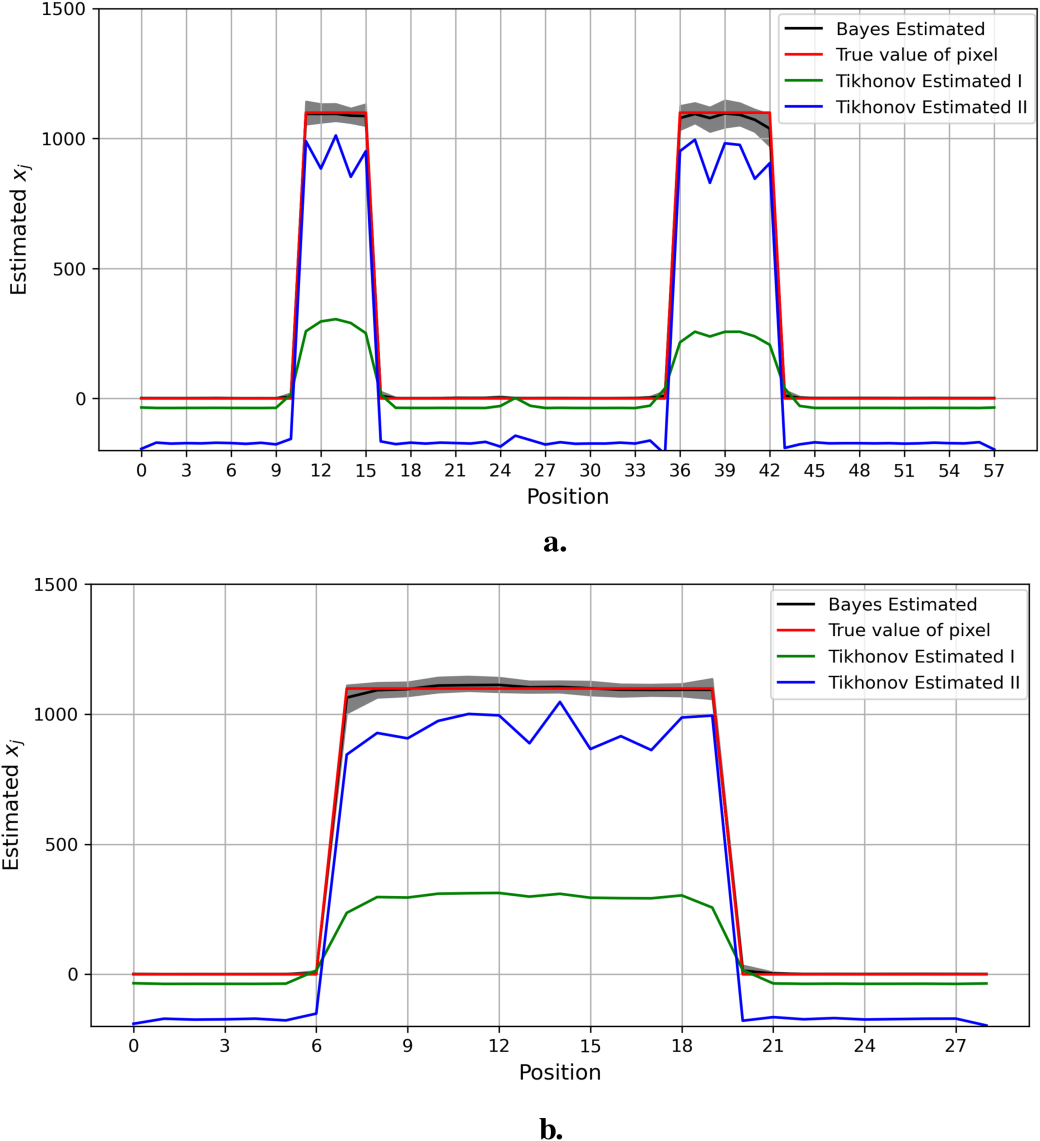
Estimation comparison between Bayesian modeling and Tikhonov regularization. The estimation from Bayesian modeling highlighted in the dark color has a credible interval in gray. The estimations from Tikhonov regularization are presented with two regularization options: the green line represents the optimum regularization defined by the cross-validation method, while the blue line represents manual regularization with λ=0.1 applied. Here, the pixel estimations for the 20th row are shown at the top, while those for the 36th column are shown at the bottom. **(a)** Estimation comparison between Bayesian modeling and Tikhonov regularization I. **(b)** Estimation comparison between Bayesian modeling and Tikhonov regularization II.

Similarly, the estimations of specific columns and rows within the pixel matrix from Bayesian modeling and Tikhonov regularization are presented in Figure 18. Compared to the Bayesian modeling, Tikhonov regularization is only based on the pixel point estimation without consideration of the pixels’ environment. Furthermore, unlike estimations from Markov chain Monte Carlo within the Bayesian framework, the distribution of estimated pixels and the quantified information, including the confidence intervals of estimations, are not available.

### 6.2 Real medical image application

Here, the employed medical image for the mouse kidney was obtained by using a dimercaptosuccinic acid scan (DMSA). Compared to the γ-${\text{eye}}^{TM}$ camera, the DMSA scan with technetium-99 m labeled radiotracer is well-known for its valuable capability in identifying patients’ kidney shape and location.

**Figure 19:**
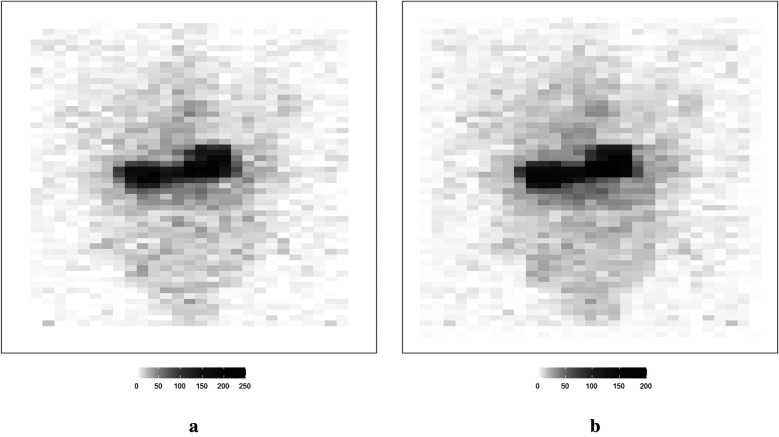
A real application in a medical image using DMSA (left) and the corresponding posterior estimations from Bayesian modeling with LMRF prior distribution (right). **(a)** Observed image from DMSA. **(b)** Estimation from Bayesian modeling.

The information of the region of interest, where the kidneys are located, is clearer in the reprocessed image in Figure 19b compared to the observed image in Figure 19a.

## 7 Conclusions

We extended the hierarchical Bayesian model for image processing by introducing the locally adaptive hyper-prior variance τ, replacing a single homogeneous hyper-prior variance τ. The locally adaptive model adjusted the hyper-prior variances based on the different local spatial conditions, effectively allowing the hyper-prior variances to vary for each location estimation. This adaptation provided the model with greater flexibility in estimation, subsequently improving accuracy. In our exploration of hyper-prior parameter estimation, we found that weakly informative prior distributions, such as a relatively flat exponential distribution, performed more efficiently compared to non-informative priors. This was evidenced by higher convergence rates and lower estimation correlations.

The locally adaptive Bayesian model with inhomogeneous hyper-prior parameters can specifically describe the probabilistic distribution of each unknown pixel. Beyond pixel-wise posterior estimation, these inhomogeneous hyper-prior variances enabled a more detailed exploration of the outcomes. For instance, plotting the estimated hyper-prior variances directly revealed spatial information about the pixels. In conclusion, the locally adaptive Bayesian approach constructs a hierarchical model that encompasses multiple levels of parameters. This approach effectively integrates information from estimated parameters across different levels, leading to improved image estimation. Consequently, there is the potential for enhancements in quantification, diagnosis, and treatment monitoring in medical imaging applications.

## Data Availability

The original contributions presented in the study are included in the article/Supplementary Material, further inquiries can be directed to the corresponding authors.

## Footnotes

^1^The estimation algorithm for regularization parameter λ “cv.glmnet” and the application of Tikhonov regularization “glmnet” can be find in R package “glmnet.”

## Appendix

### Estimation algorithm of Markov chain Monte Carlo

The hyper-parameter τ is a global variable, and we assume the distribution for a global hyper-parameter τ is a uniform distribution. However, $\tau =\{\tau_{j},j=1,2,\ldots ,m\}$ becomes a vector of unknown parameters. Therefore, there are corresponding changes in terms of distribution $\pi_{\tau |\gamma }(\tau |\gamma )$ and estimation process: MCMC. We initially define the Gamma hyper-prior distribution with artificial values ξ=1 and θ=2: γ∼Gamma(ξ=1,θ=2). If we want to estimate τ for the kth iteration in the Markov chain Monte Carlo and update unknown parameters, follow the steps below:

Once the chain has burned in, a process to drop a bunch of initial iterations whose value probabilities are low, the estimation for each parameter is supposed to be the sampling mean, we seek, i.e., $E(\gamma )\approx (\sum_{k}\gamma^{\left(k\right)})/k$.

### Estimation measurement

The estimation $X_{j}$ can be assessed by averaging the application outcomes for the last K stationary MCMC iterations. The most popular and common indexes for accurate measurement are the MSE, bias, and standard deviation (SD). Furthermore, residual sum squares (RSS), the modeling fitness measurement, is widely applied among model comparisons. For the subset of the whole parameters, the forms of measurement are seen in Table A2.

The entire image is of size m, while any image sub-region is of size R, i.e., R≤m. The average MCMC estimation is $E(x_{j})$ and ${\hat{x}}_{\;j}^{K}$ refers to the estimation of corresponding pixels in the Kth iteration. The denominator in each of the expressions varies based on the number of pixels considered.

**Table A1:** MCMC for modeling with locally adaptive hyper-parameter γ.

**Algorithm** MCMC for modelling with locally adaptive hyper-prior parameters (iteration k)
**Input**: A list of initial values $\left\{X_{1}=x_{1}^{\left(0\right)},\;X_{2}=x_{2}^{\left(0\right)},\;\ldots ,\;X_{m}=x_{m}^{\left(0\right)}\right\}$;
A list of initial values $\left\{\tau_{1}=\tau_{1}^{\left(0\right)},\;\tau_{2}=\tau_{2}^{\left(0\right)},\;\ldots ,\;\tau_{m}=\tau_{m}^{\left(0\right)}\right\}$;
An updated positive constant $\sigma^{\left(k\right)}$, $\sigma_{\tau }^{\left(k\right)}$,$\sigma_{\gamma }^{\left(k\right)}$.
**For** j={1,2,…,m}
1. Propose a new value $x_{\;j}^{\left(k\right)}∼N(x_{\;j}^{\left(k-1\right)},{\left(\sigma^{\left(k\right)}\right)}^{2})$; **if and only if** $x_{\;j}^{k}\geq 0$
2. Generate μ∼unif(0,1)
3. Accept $x_{j}^{k}$ with probability
$\alpha =\text{min}\left(1,\frac{\;f_{X|Y,\tau }(x_{1}^{\left(k-1\right)},x_{2}^{\left(k-1\right)},\ldots ,x_{\;j}^{\left(k\right)},\ldots ,x_{m}^{\left(k-1\right)}|Y,\tau^{\left(k-1\right)})}{f_{X|Y,\tau }(x_{1}^{\left(k-1\right)},x_{2}^{\left(k-1\right)},\ldots ,x_{\;j}^{\left(k-1\right)},\ldots ,x_{m}^{\left(k-1\right)}|Y,\tau^{\left(k-1\right)})}\right)$
4. Compare the μ with the calculated α,
5. **if**: μ≤α **then**
6. Accept the proposal value $x_{j}=x_{\;j}^{\left(k\right)}$
7. **else** $x_{j}=x_{\;j}^{\left(k-1\right)}$
**end** update x
8. Propose a new candidate value $\tau_{\;j}^{k}∼N(\tau_{\;j}^{k-1},(\sigma_{\tau }^{\left(k\right)}{)}^{2})$; **if and only if $\tau_{\;j}^{k}\geq 0$**
9. Generate $\mu_{1}∼\text{unif}(0,1)$.
10. Accept $\tau^{\left(k\right)}$ with probability
$\alpha_{1}=\text{min}\left(1,\frac{\;f_{\tau |X}(\tau_{1}^{\left(k-1\right)},\tau_{2}^{\left(k-1\right)},\ldots ,\tau_{\;j}^{\left(k\right)},\ldots ,\tau_{m}^{\left(k-1\right)}|x^{k},\gamma^{\left(k-1\right)})}{f_{\tau |X}(\tau_{1}^{\left(k-1\right)},\tau_{2}^{\left(k-1\right)},\ldots ,\tau_{\;j}^{\left(k-1\right)},\ldots ,\tau_{m}^{\left(k-1\right)}|x^{k},\gamma^{\left(k-1\right)})}\right),$
11. **if**: $\mu_{1}\leq \alpha_{1}$ **then**
12. Accept the proposal value $\tau_{j}=\tau_{\;j}^{\left(k\right)}$,
13. **else** $\tau_{\;j}=\tau_{\;j}^{\left(k-1\right)}$
**end** update τ
14. Propose a new candidate value $\gamma^{k}∼N(\gamma^{k-1},(\sigma_{\tau }^{\left(k\right)}{)}^{2})$; **if and only if** $\gamma^{k}\geq 0$
15. Generate $\mu_{2}∼\text{unif}(0,1)$.
16. Accept $\gamma^{\left(k\right)}$ with probability
$\alpha_{2}=\text{min}\left(1,\frac{\;f_{\gamma |X,\tau }(\gamma^{k}|x^{k},\tau^{\left(k\right)})}{f_{\gamma |X,\tau }(\gamma^{k-1}|x^{k},\tau^{\left(k\right)})}\right),$
17. **if**: $\mu_{2}\leq \alpha_{2}$ **then**
18. Accept the proposal value $\gamma =\gamma^{\left(k\right)}$,
19. **else** $\gamma =\gamma^{\left(k-1\right)}$
**end** update γ
20. **end if**
Repeat the above steps until it receives enough sampling size.

**Table A2:** List of statistical measurements.

Measurement	Equation
Absolute bias	$(\sum_{\;j=1}^{m}|x_{\;j}-E(x_{\;j})|)/m$
Regional absolute bias	$\left(\sum_{\;j\in R}|x_{\;j}-E\left(x_{\;j}\right)|\right)/R$
Standard deviation	$\sqrt{\sum_{k=1}^{K}\sum_{\;j=1}^{m}|x_{\;j}^{\left(k\right)}-E\left(x_{j}\right){|}^{2}}/\left(K\cdot m\right)$
Regional standard deviation	$\sqrt{\sum_{k=1}^{K}\sum_{\;j\in R}|x_{\;j}^{\left(k\right)}-E\left(x_{j}\right){|}^{2}}/\left(K\cdot R\right)$
Mean square error	$\sqrt{\sum_{k=1}^{K}\sum_{\;j=1}^{m}|x_{\;j}^{\left(k\right)}-E\left(x_{j}\right){|}^{2}}/\left(K\cdot m\right)$
Regional mean square error	$\sqrt{\sum_{k=1}^{K}\sum_{\;j\in R}|x_{\;j}^{\left(k\right)}-E(x_{j}){|}^{2}}/(K\cdot R)$
Residual sum of squares	$\sum_{k=1}^{K}\sum_{\;j=1}^{m}{\left(y_{\;j}-y_{\;j}^{\left(k\right)}\right)}^{2}/\left(K\cdot m\right)$
Regional residual sum of squares	$\sum_{k=1}^{K}\sum_{\;j\in R}{\left(y_{\;j}-y_{\;j}^{\left(k\right)}\right)}^{2}/\left(K\cdot R\right)$
