# Supplementary figures and images for: Bayesian modeling with locally adaptive prior parameters in small animal imaging
Source: Front Nucl Med. 2025 Mar 4;5:1508816. doi: 10.3389/fnume.2025.1508816 (PMC11913876; doi:10.3389/fnume.2025.1508816)

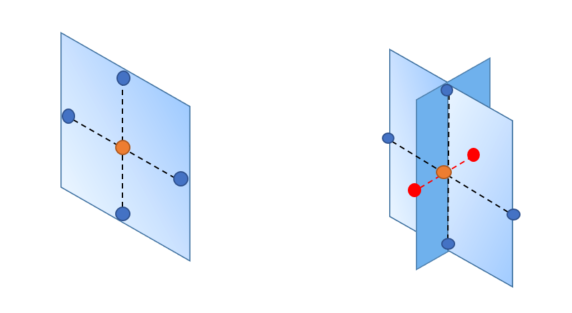

Supplement: Supplementary file 1 [file Datasheet1.zip › Figures/2d 3d display.png]

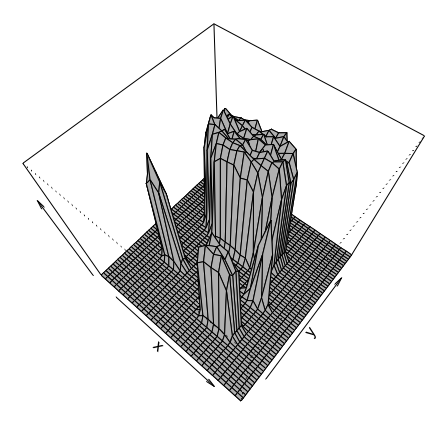

Supplement: Supplementary file 1 [file Datasheet1.zip › Figures/Applied Dataset Figures/noise simulated 3d.png]

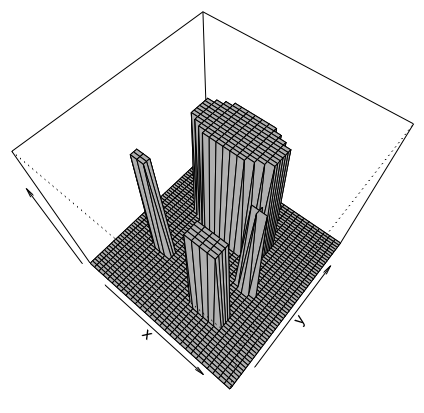

Supplement: Supplementary file 1 [file Datasheet1.zip › Figures/Applied Dataset Figures/simulated 3d.png]

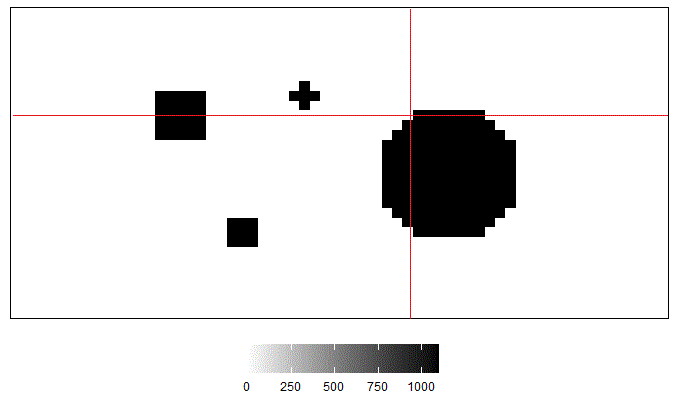

Supplement: Supplementary file 1 [file Datasheet1.zip › Figures/Applied Dataset Figures/Simulated data with true X sd=0.5.png]

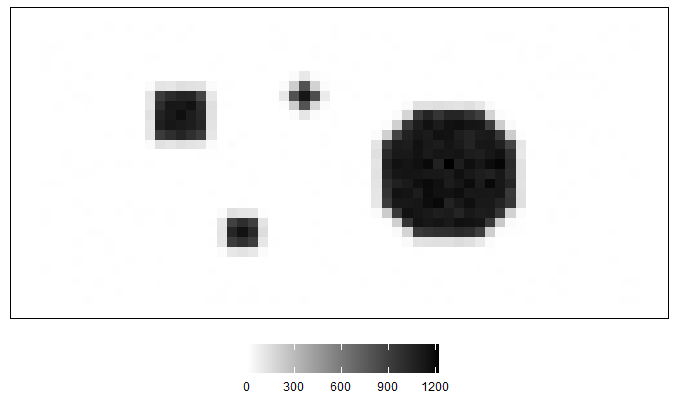

Supplement: Supplementary file 1 [file Datasheet1.zip › Figures/Applied Dataset Figures/Simulated data Y.png]

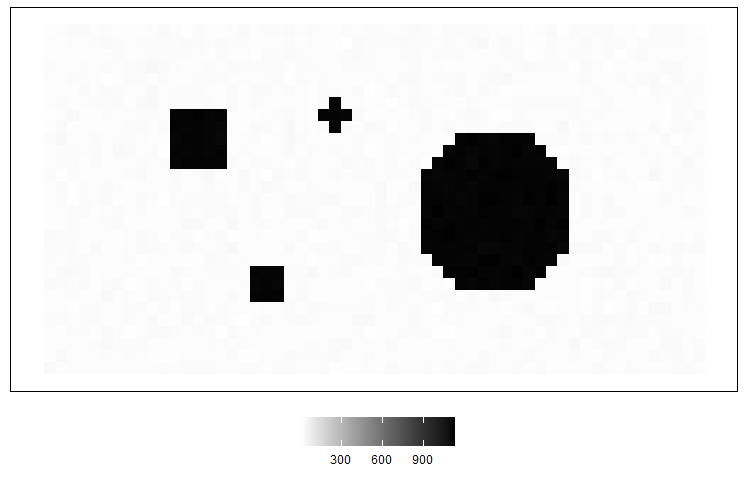

Supplement: Supplementary file 1 [file Datasheet1.zip › Figures/Applied Dataset Figures/Simulated X.png]

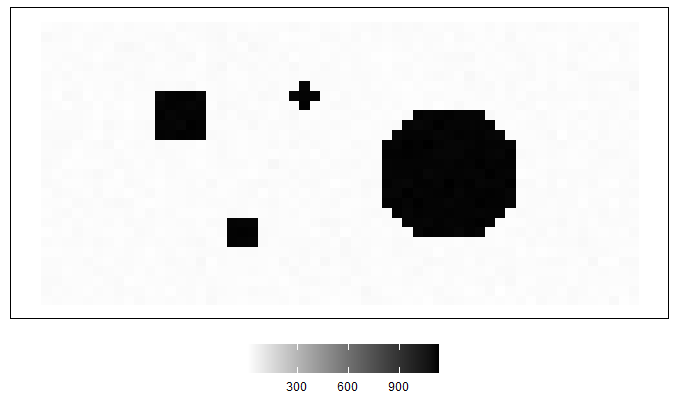

Supplement: Supplementary file 1 [file Datasheet1.zip › Figures/Applied Dataset Figures/Simulated X2.png]

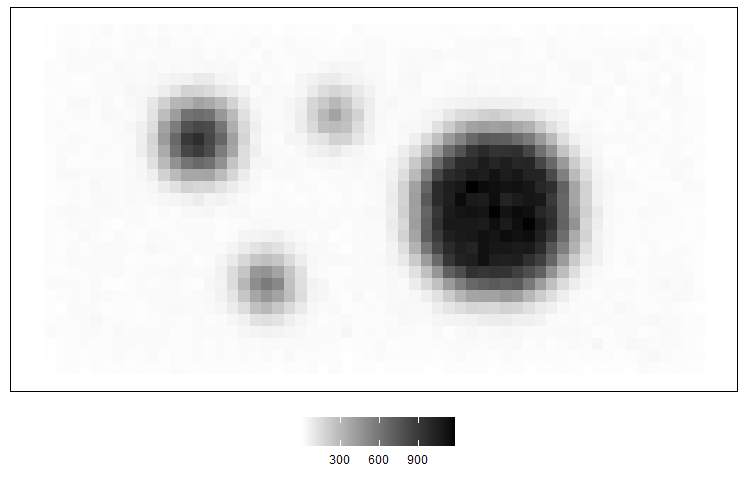

Supplement: Supplementary file 1 [file Datasheet1.zip › Figures/Applied Dataset Figures/Simulated Y.png]

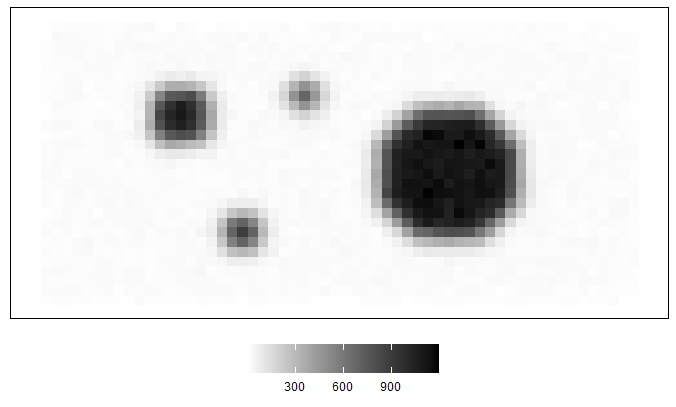

Supplement: Supplementary file 1 [file Datasheet1.zip › Figures/Applied Dataset Figures/Simulated Y2.png]

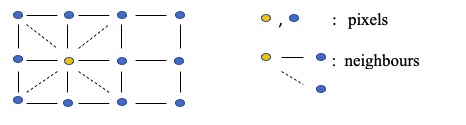

Supplement: Supplementary file 1 [file Datasheet1.zip › Figures/lattice pattern.jpg]

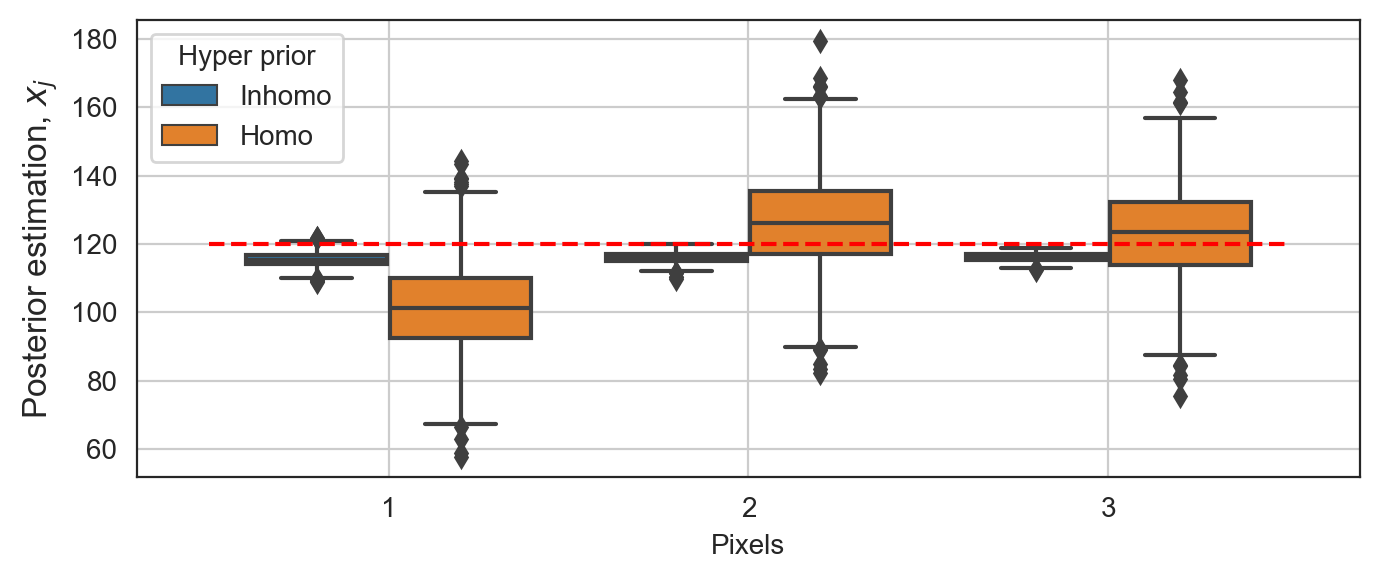

Supplement: Supplementary file 1 [file Datasheet1.zip › Figures/Locally adaptive prior parameters/estimation boxplot comparison hyper vs homo background.png]

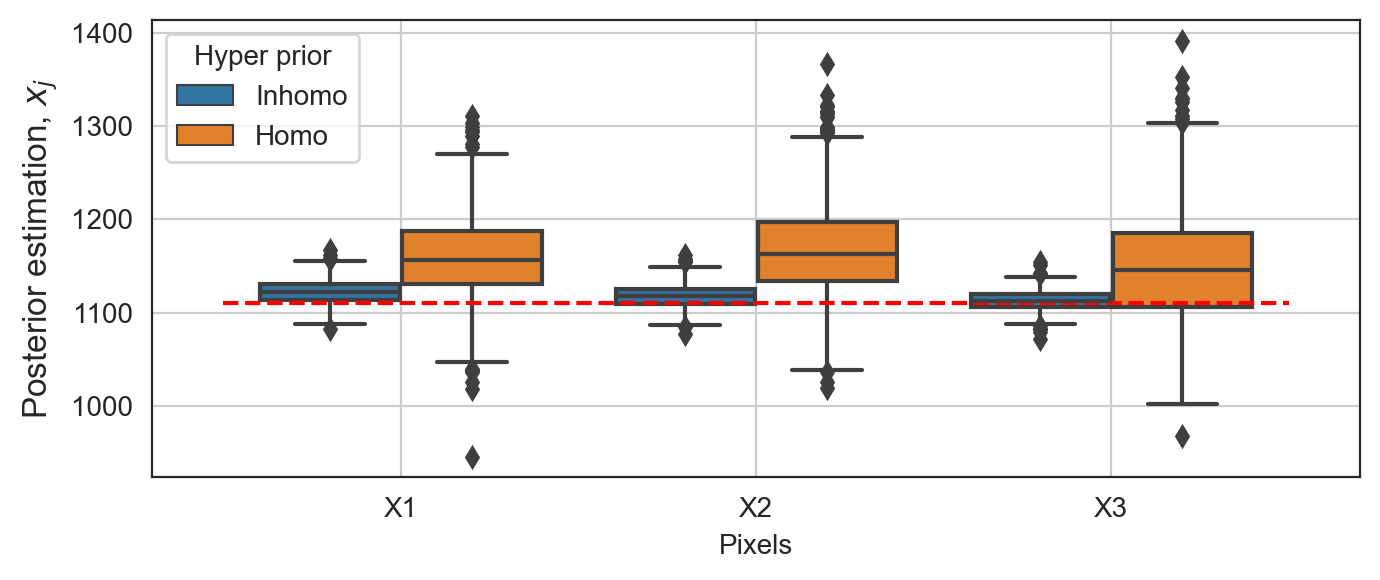

Supplement: Supplementary file 1 [file Datasheet1.zip › Figures/Locally adaptive prior parameters/estimation boxplot comparison hyper vs homo.png]

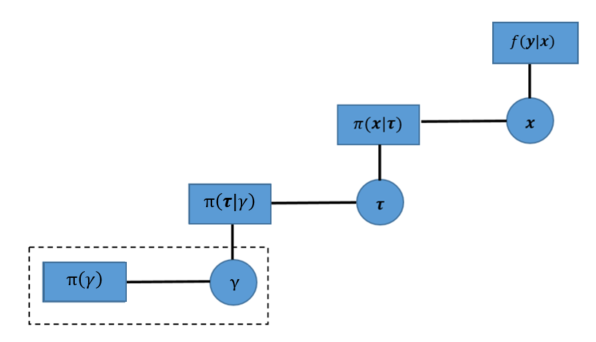

Supplement: Supplementary file 1 [file Datasheet1.zip › Figures/Locally adaptive prior parameters/factor display for hyper parapeter in hyper piror distribution.png]

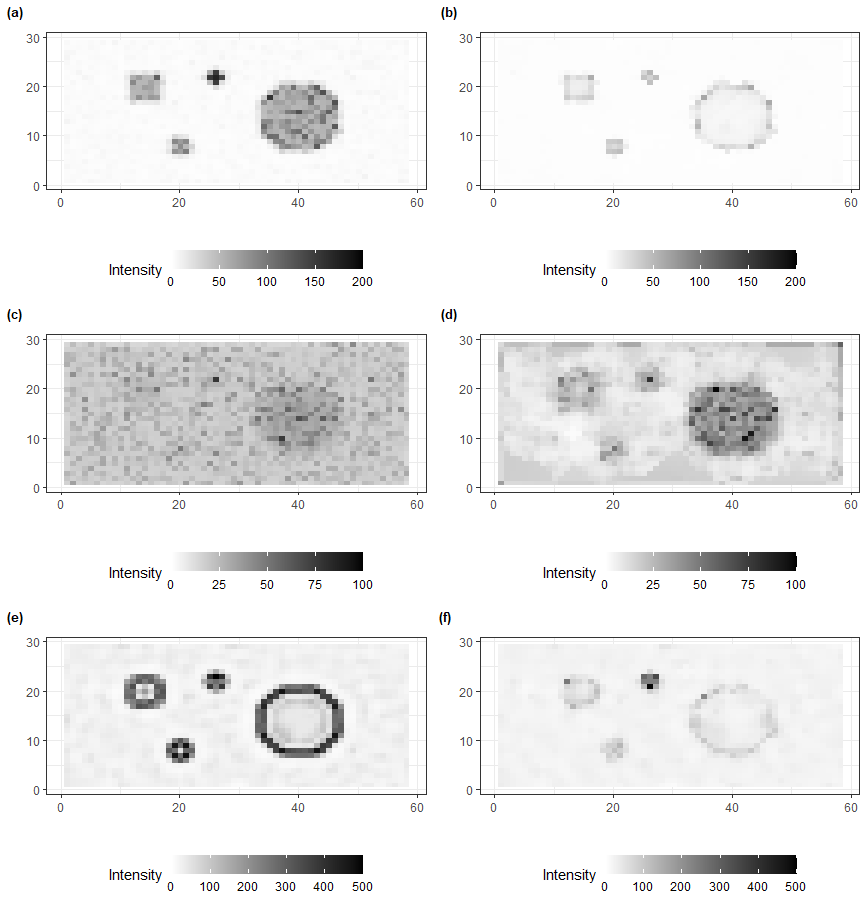

Supplement: Supplementary file 1 [file Datasheet1.zip › Figures/Locally adaptive prior parameters/homo vs hyper bias.png]

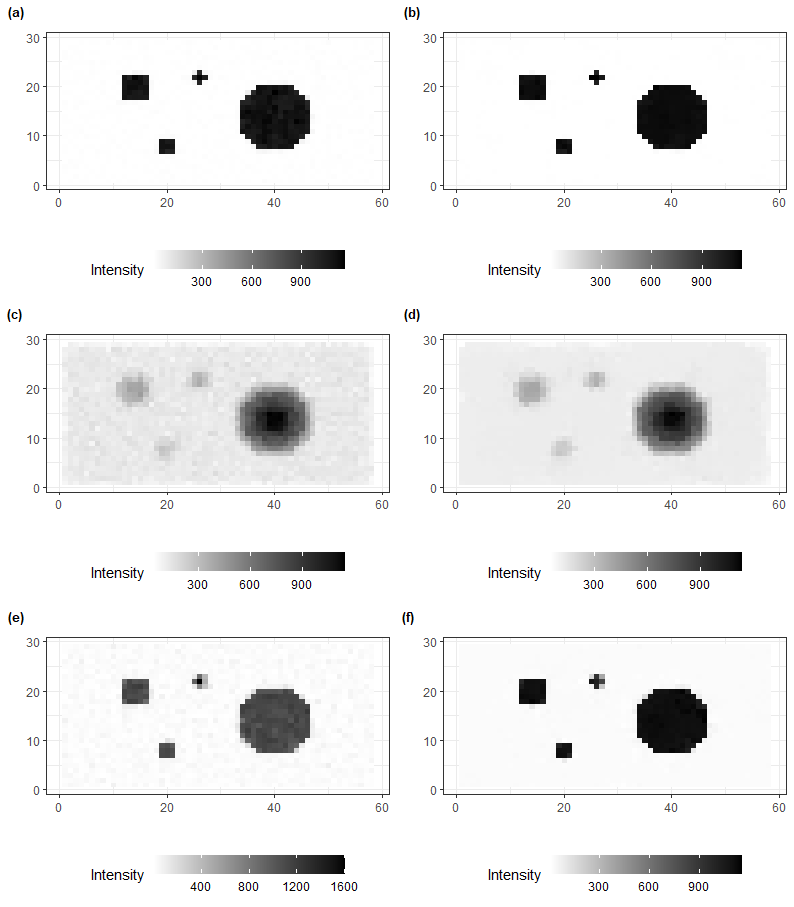

Supplement: Supplementary file 1 [file Datasheet1.zip › Figures/Locally adaptive prior parameters/homo vs hyper parameter.png]

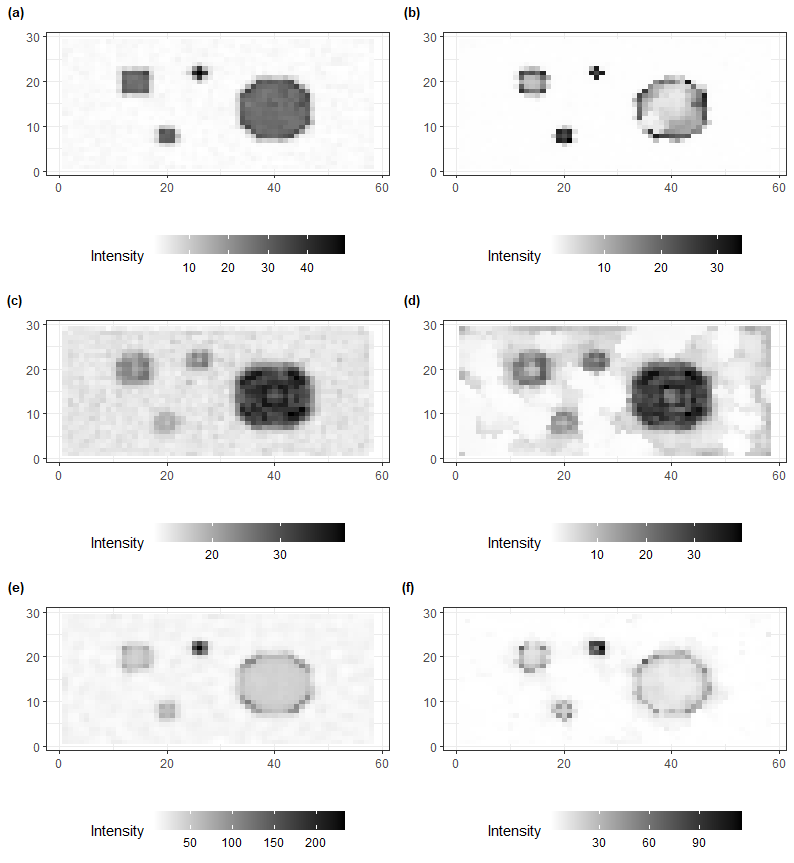

Supplement: Supplementary file 1 [file Datasheet1.zip › Figures/Locally adaptive prior parameters/homo vs hyper sd.png]

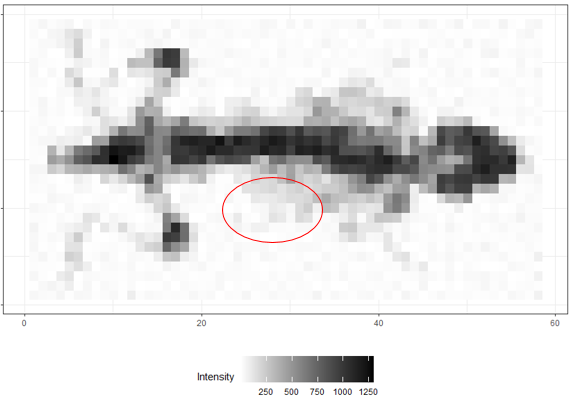

Supplement: Supplementary file 1 [file Datasheet1.zip › Figures/Locally adaptive prior parameters/homo_mouse.png]

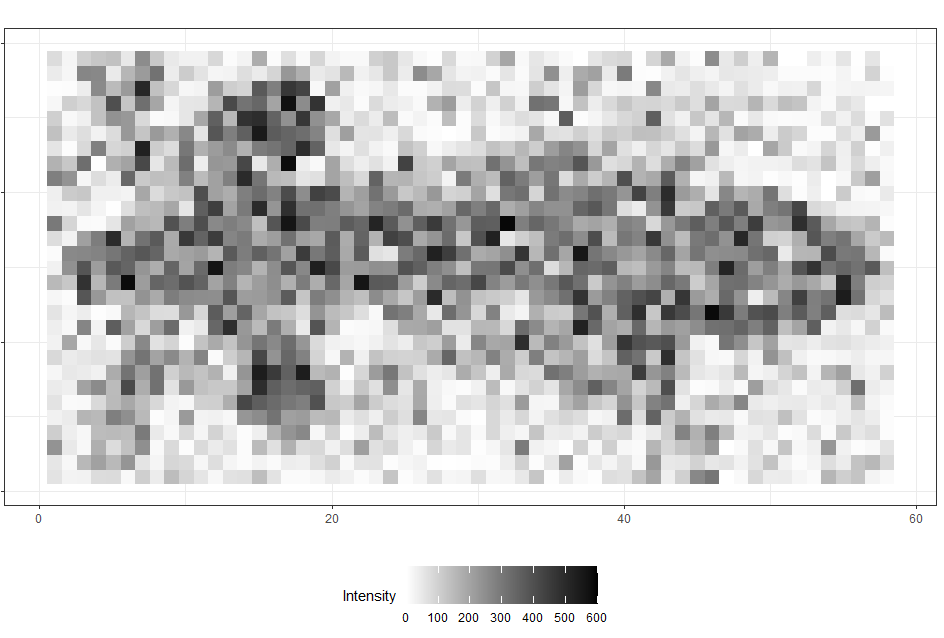

Supplement: Supplementary file 1 [file Datasheet1.zip › Figures/Locally adaptive prior parameters/Hyper prior mapping mouse.png]

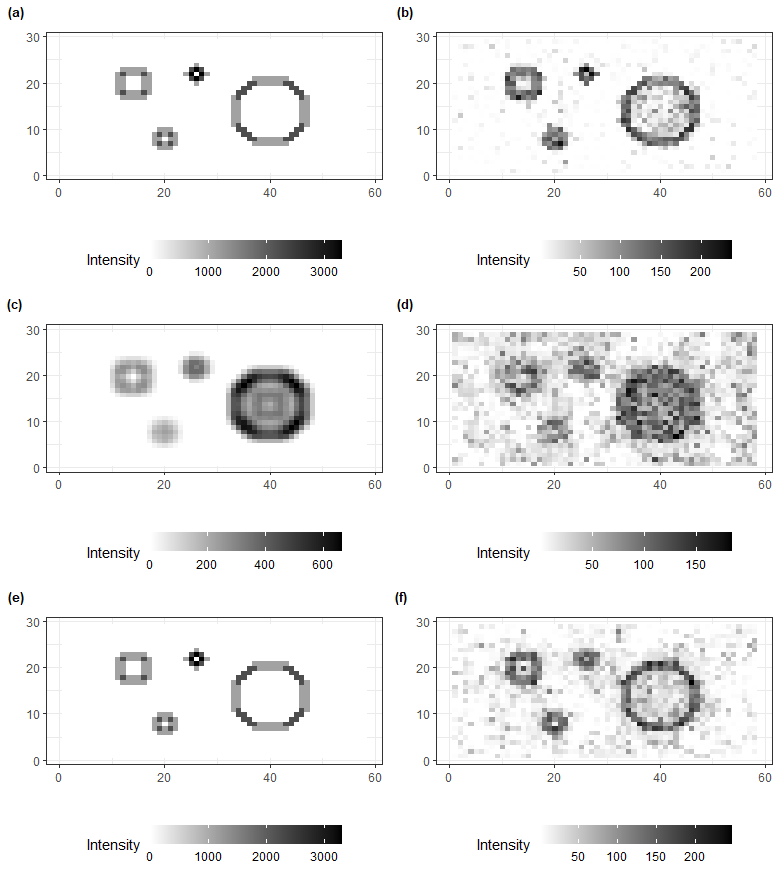

Supplement: Supplementary file 1 [file Datasheet1.zip › Figures/Locally adaptive prior parameters/Hyper prior parameters spatial information.png]

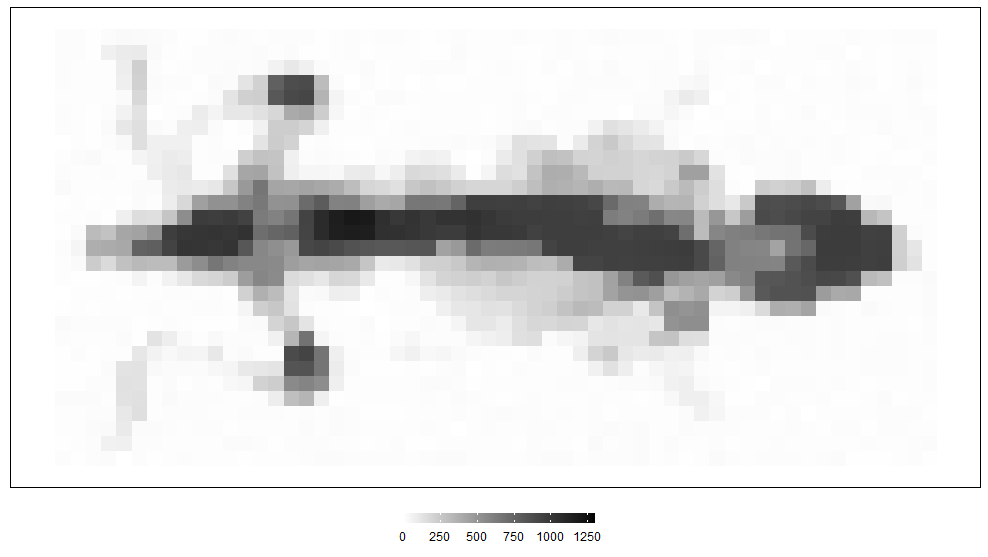

Supplement: Supplementary file 1 [file Datasheet1.zip › Figures/Locally adaptive prior parameters/Hyper prior reconstruction.png]

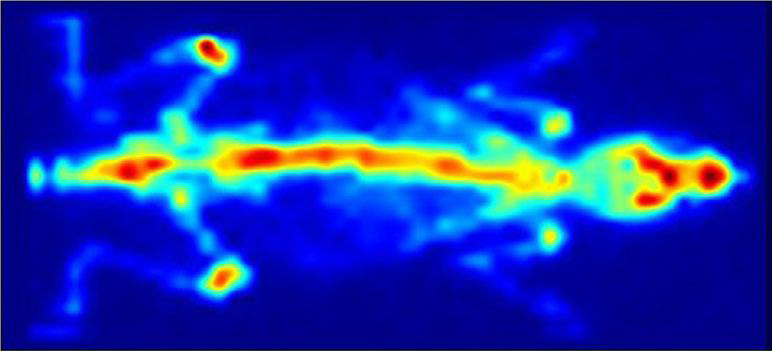

Supplement: Supplementary file 1 [file Datasheet1.zip › Figures/Locally adaptive prior parameters/mouse gamma-camera.png]

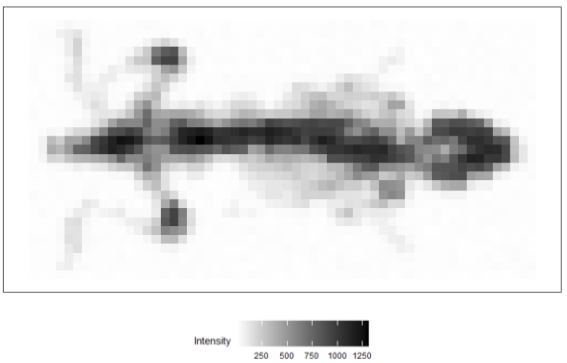

Supplement: Supplementary file 1 [file Datasheet1.zip › Figures/Locally adaptive prior parameters/mouse-observe.png]

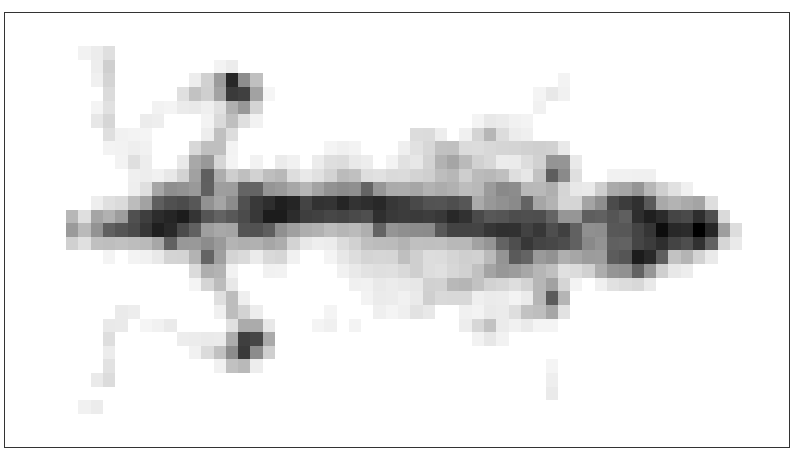

Supplement: Supplementary file 1 [file Datasheet1.zip › Figures/Locally adaptive prior parameters/mouse-observe_new.png]

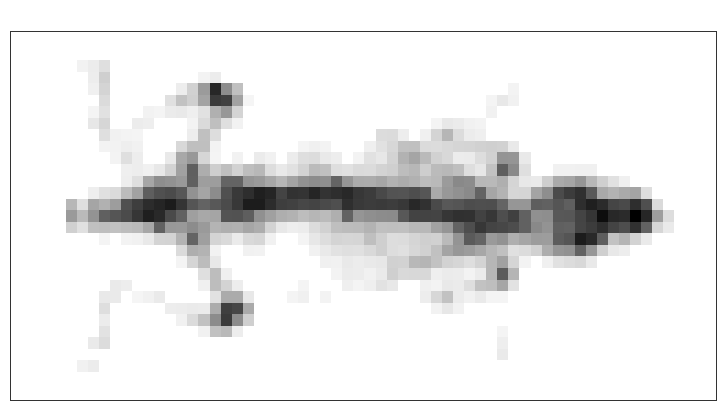

Supplement: Supplementary file 1 [file Datasheet1.zip › Figures/Locally adaptive prior parameters/mouse-simulated_new.png]

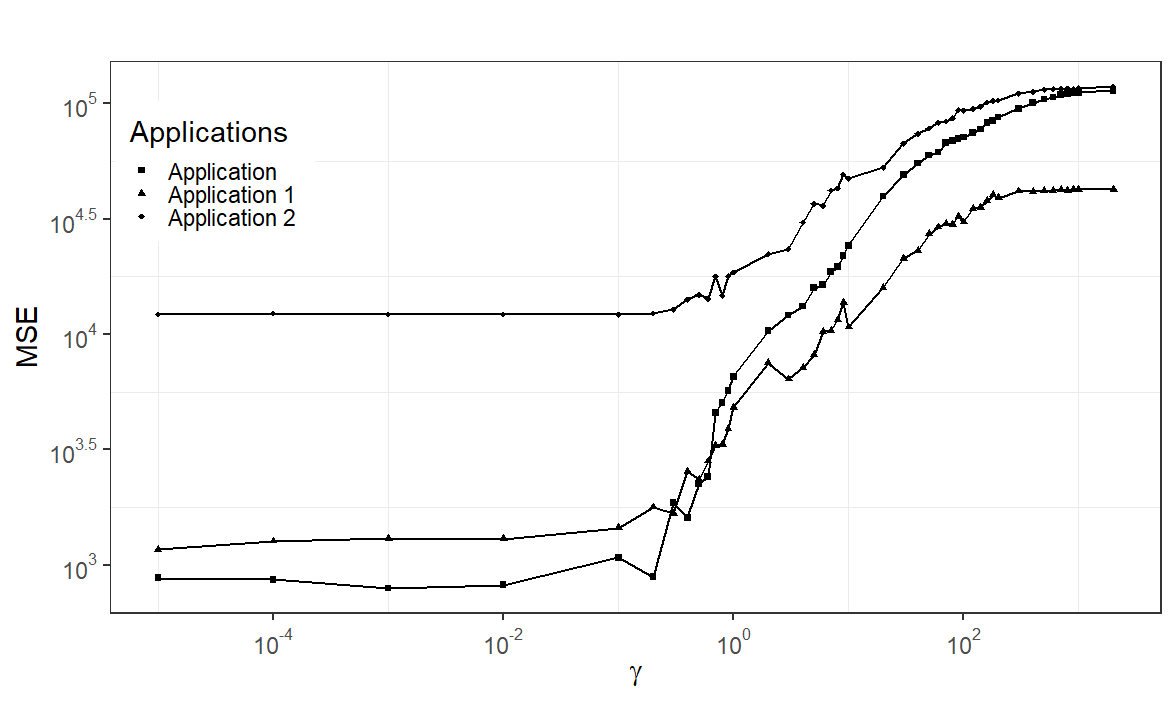

Supplement: Supplementary file 1 [file Datasheet1.zip › Figures/Locally adaptive prior parameters/MSE under the fix gamma.png]

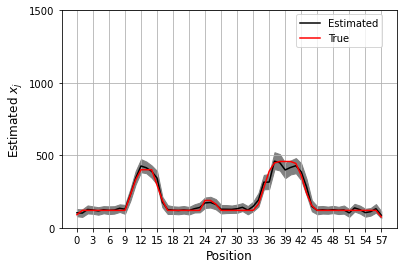

Supplement: Supplementary file 1 [file Datasheet1.zip › Figures/Locally adaptive prior parameters/row homo kernel=0.5.png]

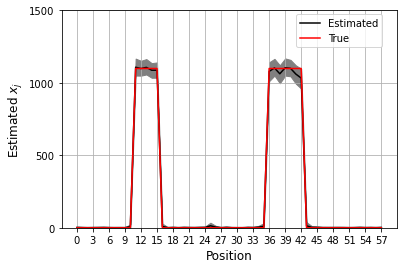

Supplement: Supplementary file 1 [file Datasheet1.zip › Figures/Locally adaptive prior parameters/row homo sd=0.5.png]

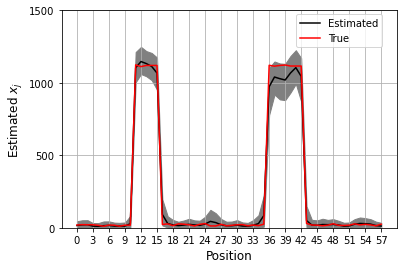

Supplement: Supplementary file 1 [file Datasheet1.zip › Figures/Locally adaptive prior parameters/row homo sd=1.png]

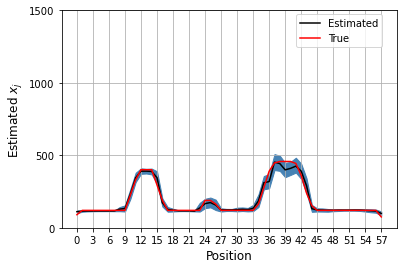

Supplement: Supplementary file 1 [file Datasheet1.zip › Figures/Locally adaptive prior parameters/row hyper kernel=0.5.png]

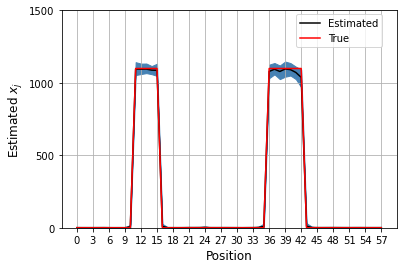

Supplement: Supplementary file 1 [file Datasheet1.zip › Figures/Locally adaptive prior parameters/row hyper sd=0.5.png]

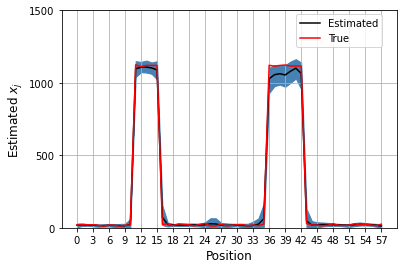

Supplement: Supplementary file 1 [file Datasheet1.zip › Figures/Locally adaptive prior parameters/row hyper sd=1.png]

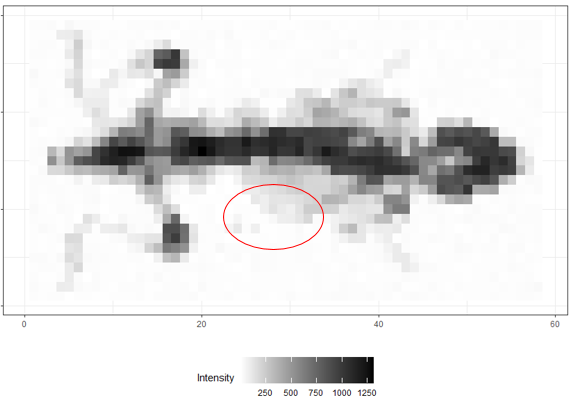

Supplement: Supplementary file 1 [file Datasheet1.zip › Figures/Locally adaptive prior parameters/simulated_mouse_laplace_hyper.png]

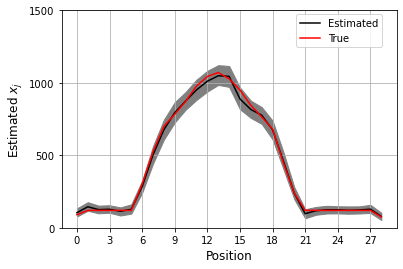

Supplement: Supplementary file 1 [file Datasheet1.zip › Figures/Locally adaptive prior parameters/vertical homo kernel=0.5.png]

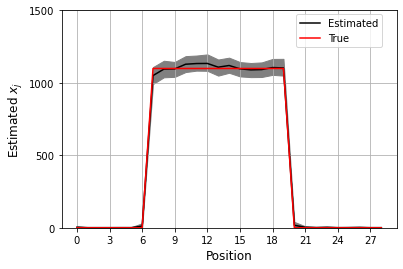

Supplement: Supplementary file 1 [file Datasheet1.zip › Figures/Locally adaptive prior parameters/vertical homo sd=0.5.png]

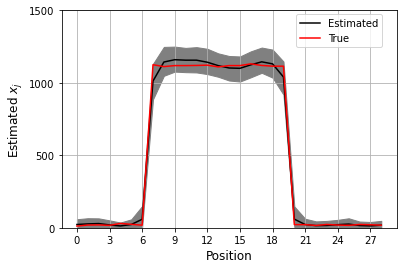

Supplement: Supplementary file 1 [file Datasheet1.zip › Figures/Locally adaptive prior parameters/vertical homo sd=1.png]

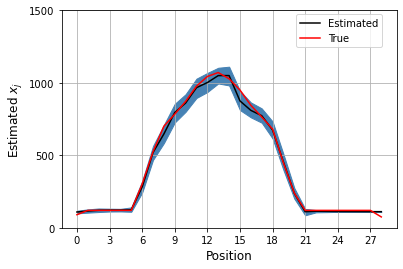

Supplement: Supplementary file 1 [file Datasheet1.zip › Figures/Locally adaptive prior parameters/vertical hyper kernel=0.5.png]

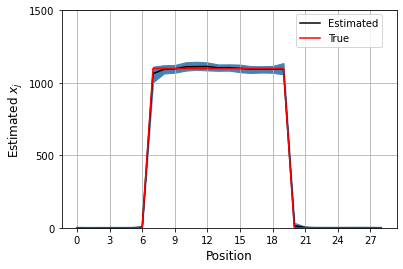

Supplement: Supplementary file 1 [file Datasheet1.zip › Figures/Locally adaptive prior parameters/vertical hyper sd=0.5.png]

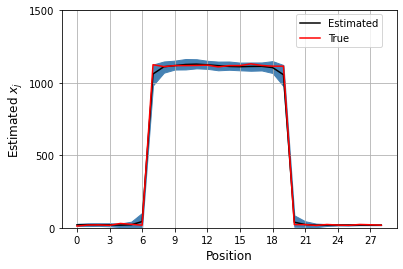

Supplement: Supplementary file 1 [file Datasheet1.zip › Figures/Locally adaptive prior parameters/vertical hyper sd=1.png]

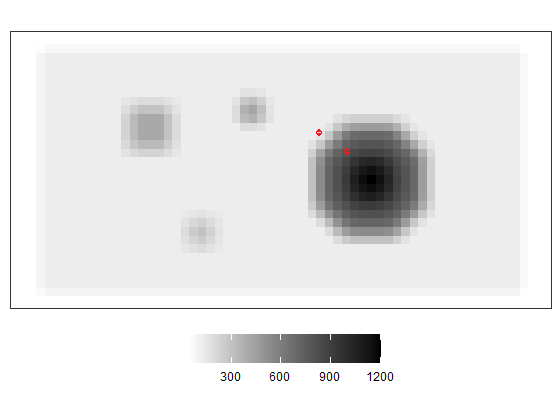

Supplement: Supplementary file 1 [file Datasheet1.zip › Figures/Smoothing datasets/Simulated X1.png]

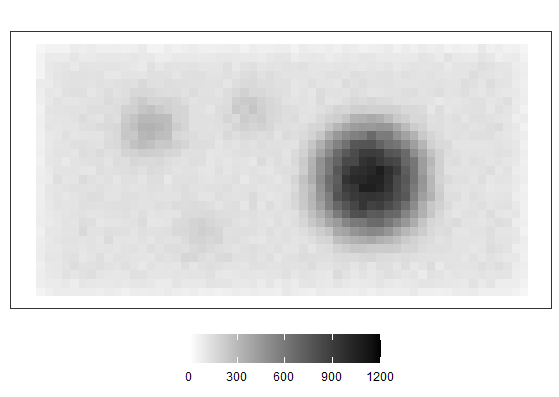

Supplement: Supplementary file 1 [file Datasheet1.zip › Figures/Smoothing datasets/Simulated Y1.png]

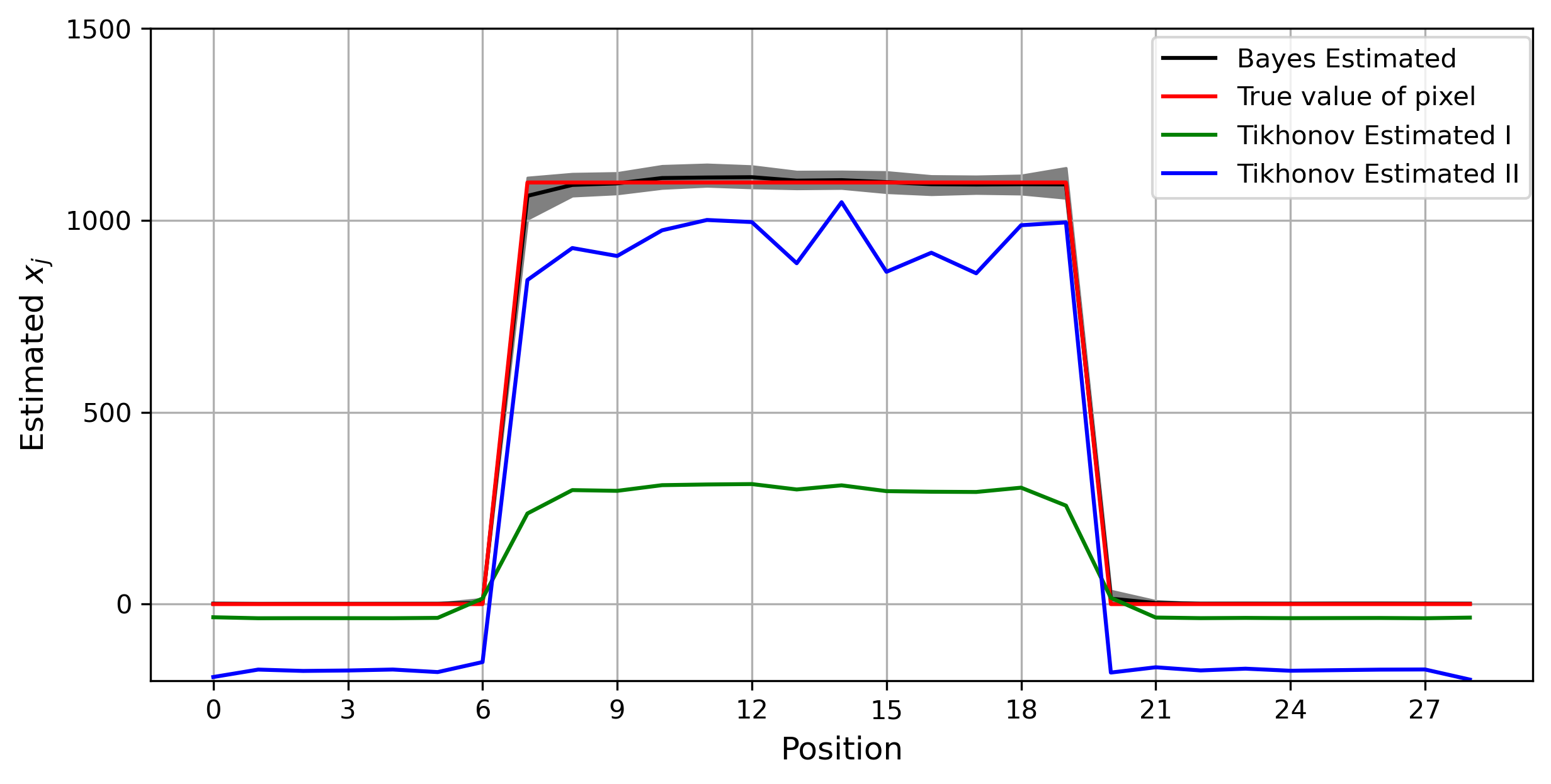

Supplement: Supplementary file 1 [file Datasheet1.zip › Figures/Tikhonov/Comparison between regulizations column.png]

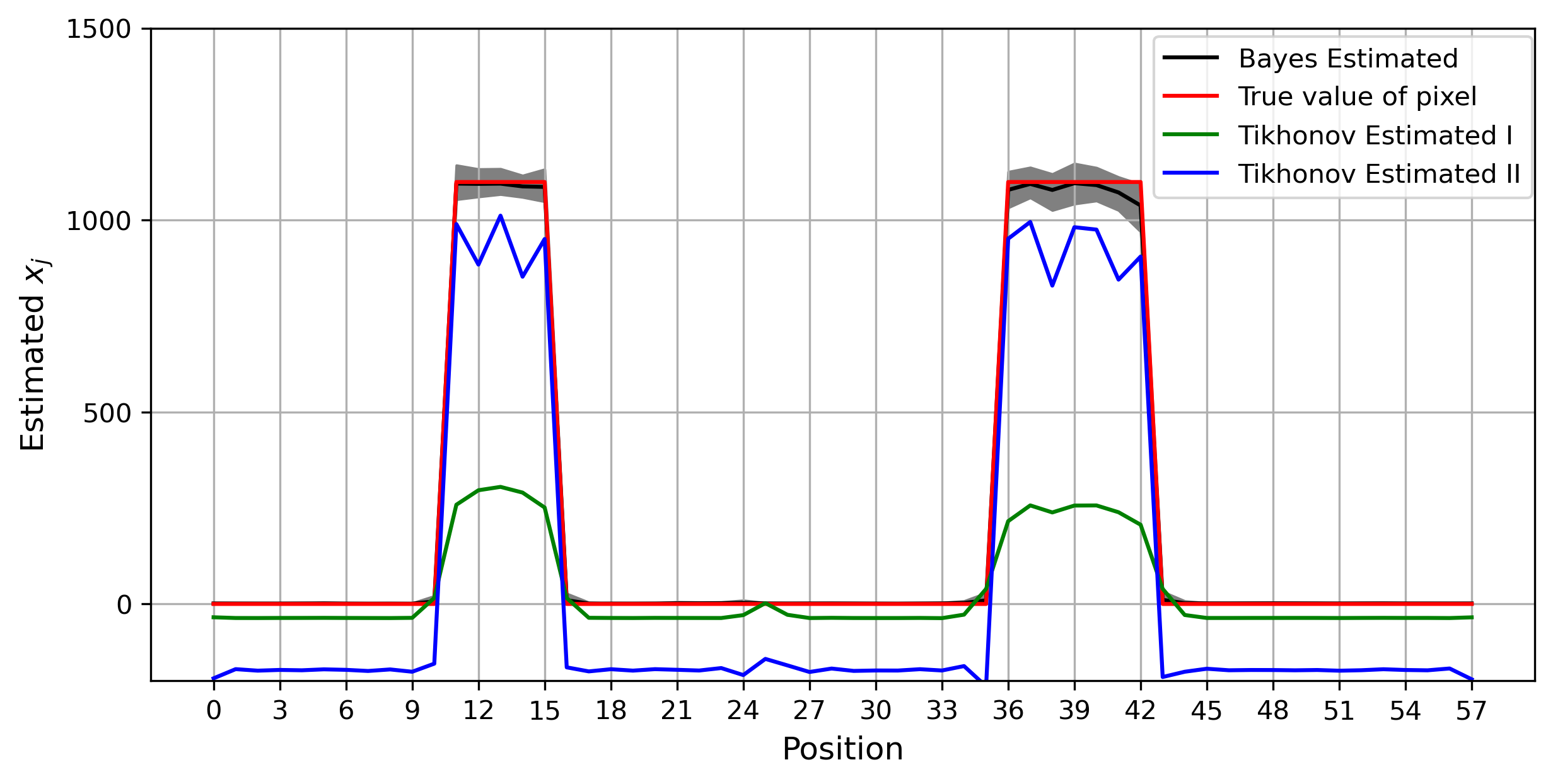

Supplement: Supplementary file 1 [file Datasheet1.zip › Figures/Tikhonov/Comparison between regulizations row.png]

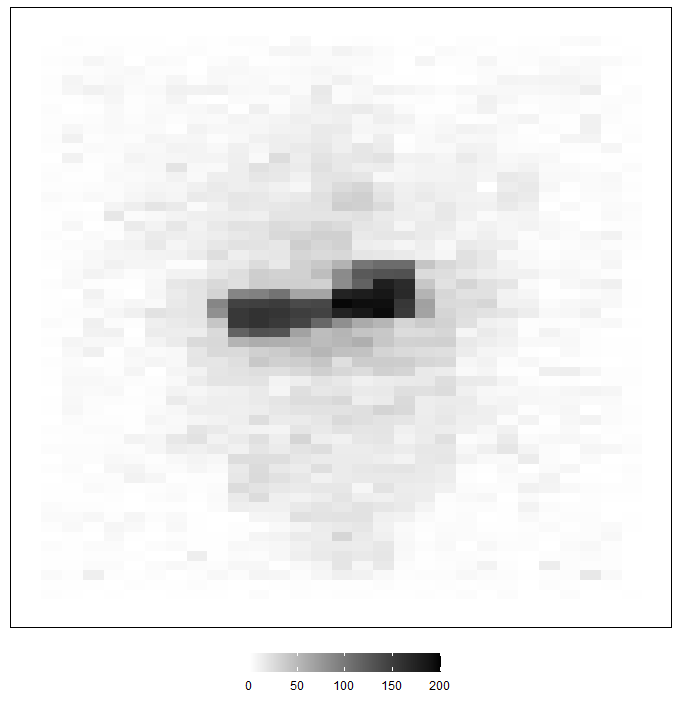

Supplement: Supplementary file 1 [file Datasheet1.zip › Figures/Tikhonov/Homogeneous application.png]

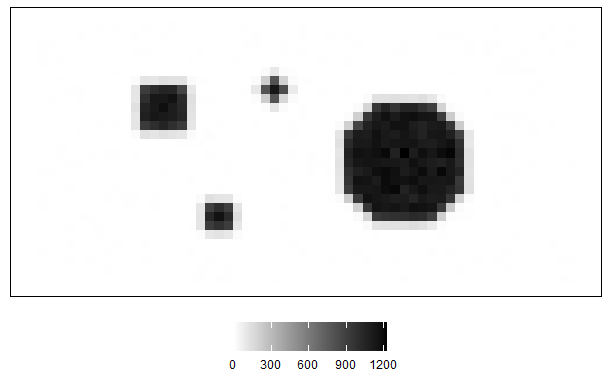

Supplement: Supplementary file 1 [file Datasheet1.zip › Figures/Tikhonov/Observation.png]

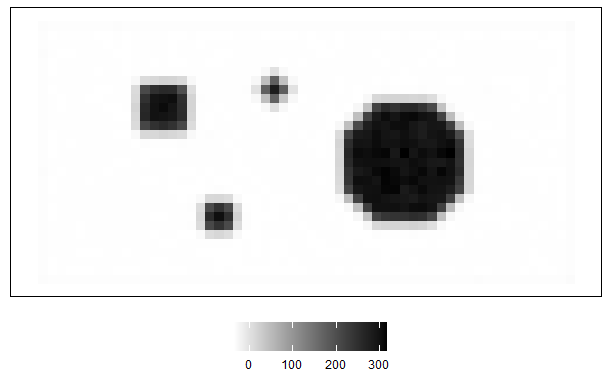

Supplement: Supplementary file 1 [file Datasheet1.zip › Figures/Tikhonov/regularization etimation.png]

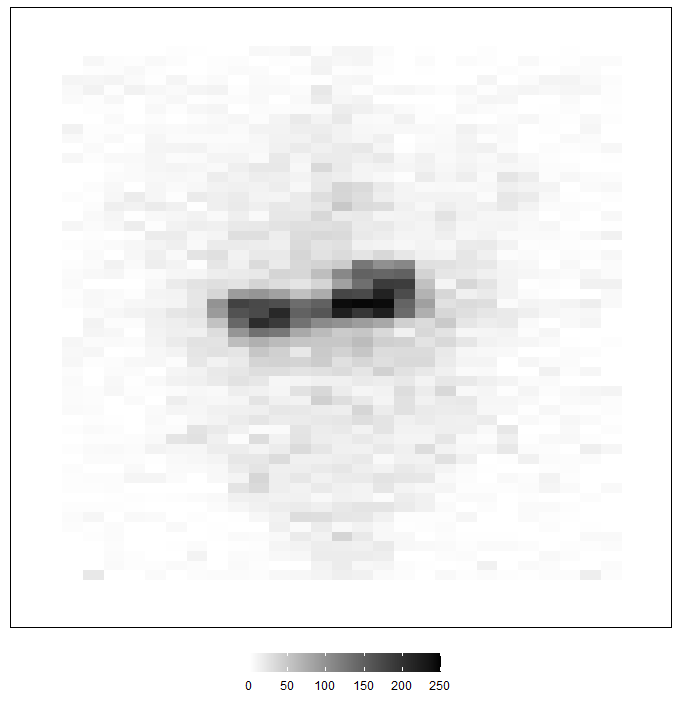

Supplement: Supplementary file 1 [file Datasheet1.zip › Figures/Tikhonov/Y observation new.png]
